# *Chromobacterium Csp_P* Reduces Malaria and Dengue Infection in Vector Mosquitoes and Has Entomopathogenic and *In Vitro* Anti-pathogen Activities

**DOI:** 10.1371/journal.ppat.1004398

**Published:** 2014-10-23

**Authors:** Jose Luis Ramirez, Sarah M. Short, Ana C. Bahia, Raul G. Saraiva, Yuemei Dong, Seokyoung Kang, Abhai Tripathi, Godfree Mlambo, George Dimopoulos

**Affiliations:** W. Harry Feinstone Department of Molecular Microbiology and Immunology, Bloomberg School of Public Health, Johns Hopkins University, Baltimore, Maryland, United States of America; Max Planck Institute for Infection Biology, Germany

## Abstract

*Plasmodium* and dengue virus, the causative agents of the two most devastating vector-borne diseases, malaria and dengue, are transmitted by the two most important mosquito vectors, *Anopheles gambiae* and *Aedes aegypti*, respectively. Insect-bacteria associations have been shown to influence vector competence for human pathogens through multi-faceted actions that include the elicitation of the insect immune system, pathogen sequestration by microbes, and bacteria-produced anti-pathogenic factors. These influences make the mosquito microbiota highly interesting from a disease control perspective. Here we present a bacterium of the genus *Chromobacterium* (*Csp_P*), which was isolated from the midgut of field-caught *Aedes aegypti*. *Csp_P* can effectively colonize the mosquito midgut when introduced through an artificial nectar meal, and it also inhibits the growth of other members of the midgut microbiota. *Csp_P* colonization of the midgut tissue activates mosquito immune responses, and *Csp_P* exposure dramatically reduces the survival of both the larval and adult stages. Ingestion of *Csp_P* by the mosquito significantly reduces its susceptibility to *Plasmodium falciparum* and dengue virus infection, thereby compromising the mosquito's vector competence. This bacterium also exerts *in vitro* anti-*Plasmodium* and anti-dengue activities, which appear to be mediated through *Csp_P* -produced stable bioactive factors with transmission-blocking and therapeutic potential. The anti-pathogen and entomopathogenic properties of *Csp_P* render it a potential candidate for the development of malaria and dengue control strategies.

## Author Summary

The infectious agents that cause malaria and dengue are transmitted by *Anopheles* and *Aedes* mosquitoes, respectively. Bacteria found in the mosquito midgut have the potential to dramatically affect the susceptibility of the mosquito vector to the malaria parasite and dengue virus. In this work, we investigate one such microbe, *Chromobacterium sp. (Csp_P)*, a bacterium isolated from a field-caught *Aedes aegypti* mosquito. We show that *Csp_P* can effectively colonize the midguts of *Anopheles gambiae* and *Aedes aegypti* mosquitoes and can, when ingested by the mosquito, significantly reduce the mosquito's susceptibility to infection with the malaria parasite and dengue virus. We also show that exposure to, and ingestion of, *Csp_P* can reduce the lifespan of larval and adult mosquitoes, respectively. We show that *Csp_P* has anti-*Plasmodium* and anti-dengue activity independent of the mosquito, suggesting that the bacterium secretes metabolites that could potentially be exploited to prevent disease transmission or to treat infection.

## Introduction

The influence of the gut microbiota on the vector competence of disease vectors such as mosquitoes has gained increasing interest over the past decade [1]–[3]. Previous work has shown that co-infection of *Anopheles* mosquitoes with *Plasmodium* and with *Serratia sp.* or *Enterobacter sp.* bacteria leads to reduced *Plasmodium* infection [3], [4]. Additionally, the presence of certain bacterial species in *Aedes* mosquito midguts leads to a lower intensity of dengue virus infection [5]. Studies have also shown that *Anopheles* and *Aedes* mosquitoes that have had their gut microbiota experimentally reduced via antibiotic treatment show higher *Plasmodium* and dengue virus infection levels, respectively, than do their untreated counterparts [6]–[8]. The anti-pathogen activity of mosquito midgut bacteria has been attributed to the elicitation of the mosquito immune system in some instances, and to direct anti-pathogenic activity of bacteria-produced molecules in others [9]. Activation of the IMD pathway, the major anti-*P. falciparum* immune pathway, has been shown to be mediated through an interaction between the pattern recognition receptor PGRP-LC and the midgut microbiota [10]. In turn, microbe-derived anti-pathogen factors have been characterized in some microbe-host interaction systems and include cytotoxic metalloproteases, hemolysins, antibiotics, haemaglutinins, proteases, prodigiosin pigments, and iron chelators (siderophores) [9].

In nature, bacteria commonly grow attached to surfaces in complex matrices of cells, proteins, polysaccharides, and DNA (biofilm growth), rather than as single free-swimming cells (planktonic growth) [11], [12]. Biofilm formation allows the bacteria to survive exposure to host-derived antimicrobial factors and other environmental stressors [11], [12]. Furthermore, bacterial cells in a biofilm have quite different gene expression and metabolic profiles than do cells in a free-swimming planktonic state [11]. Studies of *Pseudomonas aeruginosa* colonization of the *Drosophila melanogaster* gut have shown that biofilm formation can dramatically affect dissemination in the hemolymph and fly mortality [13].

In this study, we show that a *Chromobacterium sp.* isolate, *Csp_P*, previously isolated from the midgut of field-collected *Ae. aegypti* mosquitoes [5], exerts *in vitro* anti-*Plasmodium* and anti-dengue activity when grown under biofilm conditions. *Csp_P* can effectively colonize the intestines of the two most important mosquito disease vectors, *An. gambiae* and *Ae. aegypti*, where it blocks *Plasmodium* and dengue infection. It also exerts entomopathogenic activity against both larval and adult stages and could therefore be used for the development of a biocontrol agent. *Csp_P*'s anti-pathogen activities appear to be mediated by stable secondary metabolites, suggesting that *Csp_P* is a source of potentially interesting candidates for the development of therapeutic and transmission-blocking drugs.

## Results/Discussion

In a previous study, we isolated a Gram-negative bacterium *Chromobacterium sp.* (*Csp_P*) from the midgut of field-collected *Ae. aegypti* mosquitoes in Panama [5]. The genus *Chromobacterium spp.* represents soil- and water-associated bacteria of tropical and subtropical regions [14], and members of this genus are known to produce a variety of bioactive compounds [14], [15] and to form biofilms. The most extensively studied member, *Chromobacterium violaceum*, has been found to produce violacein, a violet pigment compound with potent antimicrobial, antiparasitic, and tumoricidal activity [14], [16]. *Csp_P* can be cultured in Luria Bertani (LB) broth and on LB agar, on which it forms flat colonies with a tan color that become darker with time and are opaque when exposed to light. *Csp_P* does not produce violacein, but molecular characterization of its 16s rRNA gene sequence and phylogenetic analysis showed a 98% similarity to *Chromobacterium haemolyticum* and *Chromobacterium aquaticum*, probably its two closest relatives.

### *Csp_P* colonization of the mosquito midgut

**Figure 1:**
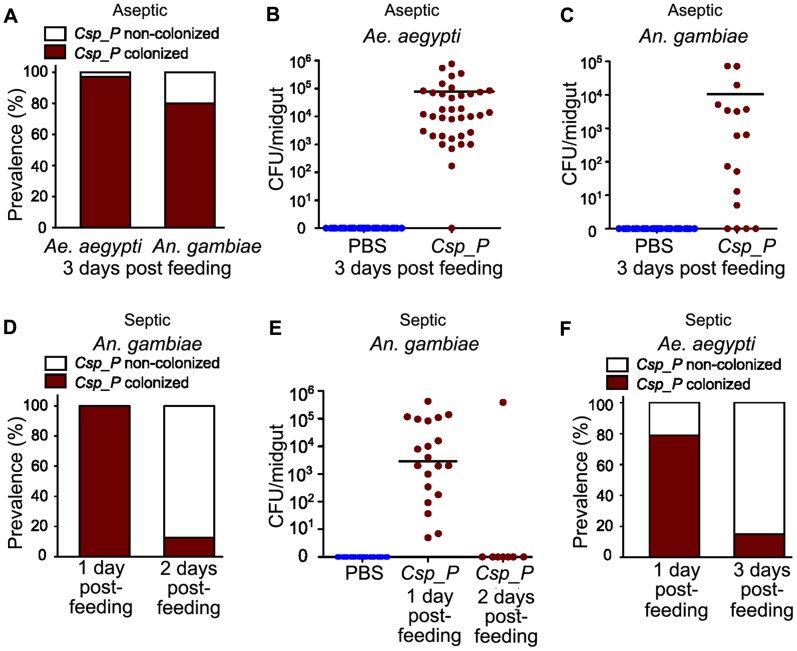
*Csp_P* colonization of the mosquito midgut. All mosquitoes were exposed to *Csp_P* via sugar meal. To introduce *Csp_P* via sugar meal, adults were allowed to feed for 24 h on 1.5% sucrose containing *Csp_P* liquid culture at a final concentration of ∼10^8^ CFU/ml for *An. gambiae* and ∼10^6^ (A, B) or 10^10^ (F) CFU/ml for *Ae. aegypti*. For antibiotic treated mosquitoes, the prevalence of *Csp_P* was measured in *Ae. aegypti* and *An. gambiae* midguts at 3 days post-exposure (A). The number of colony forming units (CFUs) of *Csp_P* was also measured in the midguts of (B) *Ae. aegypti* and (C) *An. gambiae* 3 days after exposure to *Csp_P*. Experiments for antibiotic treated *Ae. aegypti* and *An. gambiae* were replicated at least three times. Final sample sizes: n_Ae. aegypti/PBS_ = 37; n_Ae. aegypti/Csp_P_ = 37; n_An. gambiae/PBS_ = 30; n_An. gambiae/Csp_P_ = 17. For septic (*i.e.* non-antibiotic treated) mosquitoes, the prevalence and bacterial load of *Csp_P* was measured in *An. gambiae* midguts at 1 and 2 days post exposure (D,E). Experiments for septic *An. gambiae* were replicated twice. Final sample sizes: n_An. gambiae/PBS_ = 30; n_An. gambiae/Csp_P/Day 1_ = 20; n_An. gambiae/Csp_P/Day 2_ = 8. Prevalence of *Csp_P* was measured in *Ae. aegypti* midguts at 1 and 3 days post exposure (F). Experiments for septic *Ae. aegypti* were replicated twice. Final sample sizes: n_Ae. aegypti/Csp_P/Day 1_ = 19; n_Ae. aegypti/Csp_P/Day 3_ = 20. Horizontal lines indicate mean values. The following transformation was applied to all raw CFU data: y = log_10_(x+1), where x = original CFU count and y = plotted data values.

To assess the ability of *Csp_P* to colonize the mosquito midgut, we exposed antibiotic-treated mosquitoes to a sugar source containing10^6^ colony forming units (CFU)/ml for *Ae. aegypti* or 10^8^ CFU/ml for *An. gambiae* for 24 h and then dissected, homogenized and plated the midguts on LB agar plates at 3 days post-exposure. Treatment with antibiotics through the sugar meal was performed to remove the native microbial flora which can fluctuate in terms of load and species composition between individual mosquitoes of the same cage and generation, thereby complicating the interpretation of our data [7]. The presence of the native microbiota would also render it difficult to discriminate the *Csp_P* colonies from those of other species through visual inspection. *Csp_P* displayed an exceptional ability to rapidly colonize mosquito midguts, showing a prevalence of 80% in *An. gambiae* and 97% in *Ae. aegypti* cage populations at 3 days after exposure (Fig. 1A). Average bacterial loads at this time point were approximately 10^5^ and 10^4^ CFU per midgut in *Ae. aegypti* (Fig. 1B) and *An. gambiae* (Fig. 1C) females, respectively.

**Figure 2:**
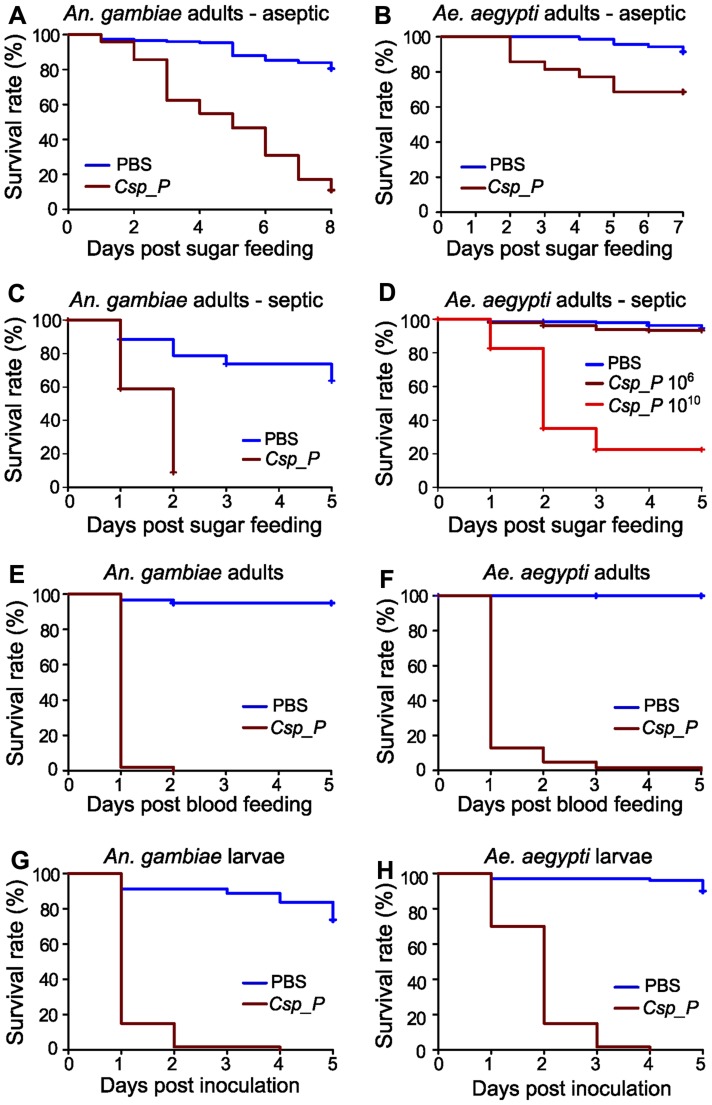
*Csp_P* exposure causes high mortality in adults and larvae. *Csp_P* was experimentally introduced into the adult midgut via either a sugar meal (A–D) or blood meal (E, F), and mortality was observed over 5–8 days. To introduce *Csp_P* via sugar meal, adults were allowed to feed for 24 h on 1.5% sucrose containing *Csp_P* liquid culture at a final concentration of ∼10^8^ CFU/ml for *An. gambiae* and ∼10^6^ or 10^10^ CFU/ml for *Ae. aegypti*. *Csp_P* ingestion significantly decreased survival in sugar-fed aseptic (*i.e.* pre-treated with antibiotics) *An. gambiae* (A, p<0.0001) and *Ae. aegypti* (B, p<0.0001). Each experiment was replicated three times. Total sample sizes: (A)_PBS_ = 149; (A)_Csp_P_ = 146; (B)_PBS_ = 70; (B)_Csp_P_ = 70. Ingestion of *Csp_P* significantly decreased survival in sugar-fed septic (*i.e.* not treated with antibiotics) *An. gambiae* (C, p<0.0001). In septic *Ae. aegypti*, survival was significantly decreased after feeding on a 10^10^ CFU/ml sugar meal (D, p<0.0001) but not after feeding on a 10^6^ CFU/ml sugar meal (D, p = 0.08). Experiments in C and D were replicated twice. Total sample sizes: (C)_PBS_ = 95; (C)_Csp_P_ = 124; (D)_PBS_ = 185; (D)_Csp10∧6_ = 223; (D)_Csp10∧10_ = 226. To introduce *Csp_P* via blood meal, *Csp_P* liquid culture (∼10^8^ CFU/ml) was mixed 1∶1 with human blood/serum and fed to septic *An. gambiae* (E) and *Ae. aegypti* (F) adults. Experiments were replicated three times with total sample sizes: (E)_PBS_ = 59; (E)_Csp_P_ = 51; (F)_PBS_ = 37; (F)_Csp_P_ = 62. The effects of *Csp_P* on larval mortality were also tested by placing 2- to 4-day-old *An. gambiae* (G) and *Ae. aegypti* (H) larvae in water containing *Csp_P* at a starting concentration of 10^6^ CFU/ml and monitoring survival over 5 days. Experiments were replicated 2–3 times with final sample sizes: (G)_PBS_ = 80; (G)_Csp_P_ = 60; (H)_PBS_ = 100; (H)_Csp_P_ = 60. P values reported above were obtained by performing pairwise Log-Rank Tests between PBS and *Csp_P* treatments. Survival curves were fitted using the Kaplan-Meier method. Vertical tick-marks indicate censored samples; in C and D multiple individuals were dissected on each day to measure *Csp_P* prevalence and bacterial load for Figure 1.

We also tested the ability of *Csp_P* to colonize the midguts of non-antibiotic treated mosquitoes. Because nearly all septic (*i.e.* non-antibiotic treated) *An. gambiae* mosquitoes had died two days after *Csp_P* introduction through sugar-feeding at 10^8^ CFU/ml (Fig. 2C), we were only able to assay prevalence and bacterial load of *Csp_P* at days one and two post feeding. At one day after *Csp_P* ingestion, we found that *Csp_P* was present in all sampled mosquitoes with an average bacterial load of 5.12×10^4^ (Figure 1D, E). At two days after *Csp_P* exposure, only 5% of *Csp_P*-fed *An. gambiae* were still alive (Fig. 2C) and *Csp_P* was detected in only one (12.5%) of these remaining mosquitoes (Fig. 1D, E). In septic *Ae. aegypti* mosquitoes that had fed on a 10^10^ CFU/ml *Csp_P*-containing sugar solution, we identified *Csp_P* in 79% of mosquitoes sampled on day 1 post feeding (Fig. 1F). At three days after feeding on the *Csp_P*-containing sugar solution, approximately 30% of the *Ae. aegypti* were still alive (Fig. 2D) and *Csp_P* was detected in 15% of these mosquitoes (Fig. 1F). These data suggest that *Csp_P* colonized the vast majority of *An. gambiae* and *Ae. aegypti* mosquitoes by day 1 post exposure and that *Csp_P* caused rapid mortality in most individuals. The small percentage that survived up to day 2 or 3, post exposure, may have received a small dose of bacteria and succeeded in clearing it by the time they were dissected. It is difficult to compare the colonization efficiency between septic and antibiotic treated mosquitoes because the survival curves differ dramatically (Fig. 2). While it appears that *Csp_P* was better at colonizing the midgut of antibiotic treated *An. gambiae* (Fig. 1A vs. 1D) and *Ae. aegypti* (Fig. 1A vs. 1F), our measurement does not take into account that individuals died much more rapidly in the septic population. This rapid mortality likely selected for mostly *Csp_P* negative individuals by day 2 and 3 post-feeding.

### *Csp_P* exerts entomopathogenic activity upon mosquito ingestion and larval exposure

We examined the influence of *Csp_P* midgut colonization on mosquito longevity by exposing antibiotic-treated *An. gambiae* and *Ae. aegypti* mosquitoes to a sugar source for 24 h containing *Csp_P* at a final concentration of 10^8^ and 10^6^ CFU/ml, respectively, and then monitoring survival. This treatment led to a decrease in the longevity of both species when compared to non-exposed control mosquitoes (Fig. 2A, B). We repeated this experiment with septic (*i.e.* not antibiotic treated) *An. gambiae* and *Ae. aegypti*. We found that feeding on a sugar source containing *Csp_P* at a concentration of 10^8^ CFU/ml resulted in rapid mortality of *An. gambiae* adult females (Fig. 2C). Mortality of septic *Ae. aegypti* females was not increased after feeding on a sugar source containing *Csp_P* at a concentration of 10^6^ CFU/ml but was dramatically increased when the sugar meal contained *Csp_P* at a concentration of 10^10^ CFU/ml (Fig. 2D). These data suggest that *Csp_P* has strong entomopathogenic activity regardless of whether other microbes are present in the mosquito gut. We observed lower survival in septic *An. gambiae* and *Ae. aegytpi* after feeding on a blood meal containing *Csp_P* at a final concentration of 10^8^ CFU/ml (Fig. 2E, F). The stronger entomopathogenic effect upon *Csp_P* introduction through the blood meal was most likely because the mosquitoes received a large single bacterial dose upon bloodfeeding rather than the multiple low doses that would be expected during sugar feeding. It is also possible that *Csp_P* proliferated to high numbers in the nutritious blood.

To study the influence of *Csp_P* on larval viability, we placed 2- to 4-day-old mosquito larvae in groups of 10 in pools containing 5 ml distilled water supplemented with 50 µl of a 1.0 OD_600_ liquid culture of *Csp_P*, and then monitored survival. This resulted in almost complete mortality of *An. gambiae* and *Ae. aegypti* larvae over a 3- and 2-day period, respectively, when compared to the control larvae that were exposed to the normal breeding water microbiota (Fig. 2G, H). These studies suggest that *Csp_P* –mediated mortality may be the direct result of a mosquitocidal factor or systemic infection through dissemination into the hemolymph; alternatively, its colonization of the midgut (or other tissues) might cause mortality indirectly by interfering with vital functions of the mosquito. Studies of *Pseudomonas aeruginosa* colonization of the *Drosophila melanogaster* gut have shown that biofilm formation can dramatically affect both dissemination within the hemolymph and fly mortality [13]. *Csp_P* is capable of forming biofilms *in vitro*, though whether biofilm formation occurs within the mosquito midgut remains untested. *C. violaceum* produces cyanide at high cell density [17], [18] via the cyanide-producing hcnABC operon, a behavior that is reportedly regulated by quorum sensing [18], [19]. Cyanide production by bacteria has been shown to cause host mortality in both nematodes [20] and insects [21]. *Chromobacterium subtsugae* has previously been shown to exert oral toxicity in various insects of agricultural importance, but not in *Culex* mosquitoes [22].

### *Csp_P* colonization of the mosquito midgut compromises pathogen infection

**Figure 3:**
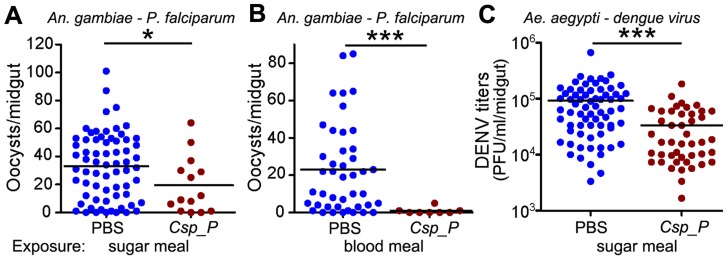
*Csp_P* reduces mosquitoes' susceptibility to malaria and dengue infection. In (A) and (C), antibiotic-treated adults were allowed to feed for 24 h on 1.5% sucrose containing *Csp_P* liquid culture at a final concentration of ∼10^8^ CFU/ml for *An. gambiae* (A) and ∼10^6^ CFU/ml for *Ae. aegypti* (C). After introduction of *Csp_P* via the sugar meal, *An. gambiae* mosquitoes were given a blood meal that contained *P. falciparum*, and *Ae. aegypti* mosquitoes were given a blood meal that contained dengue virus. In (B), *Csp_P* (10^6^ CFU/ml) was introduced concurrently with *P. falciparum* via blood meal through blood feeding of antibiotic-treated *An. gambiae*. In all experiments, PBS was used as the non-*Csp_P*-exposed control. At 7 days after infection, midguts were dissected. Oocysts were counted in *P. falciparum*-infected *An. gambiae* females, and dengue virus titers were assayed in dengue-infected *Ae. aegypti* females by conducting standard plaque assays. Experiments were initiated using similar numbers of adult females in each treatment (A, B starting numbers = 45–50/trtmt, C starting numbers = 30–40/treatment). All experiments were replicated at least three times with final samples sizes: (A)_PBS_ = 67, (A)_Csp_P_ = 14, (B)_PBS_ = 43, (B)_Csp_P_ = 8, (C)_PBS_ = 68, (C)_Csp_P_ = 45. Differences between treatments were assessed by Mann-Whitney test (*, p<0.05; ***, p<0.001).

To investigate whether the presence of *Csp_P* in the mosquito midgut could influence the infection of *An. gambiae* with *P. falciparum* and of *Ae. aegypti* with the dengue virus DENV2, we assayed the infection of mosquitoes that had been exposed to *Csp_P* through sugar feeding 2 days prior to feeding on parasite- or virus-infected blood. Approximately one week after *An. gambiae* had fed on a *P. falciparum* gametocyte culture, parasite infection was assayed by counting oocyst-stage parasites on the basal side of the mosquito midgut. DENV2 infection of the midgut of *Ae. aegypti* was assayed through standard plaque assays 7 days after an infectious bloodmeal. All experiments were initiated using similar numbers of adult females for each treatment, but because *Csp_P* exposure causes high mortality in adults (Fig. 2), very few *Csp_P*-fed mosquitoes were still alive when the parasite and dengue infection assays were conducted. Nevertheless, we found that surviving mosquitoes exposed to *Csp_P* through sugar feeding prior to feeding on infectious blood displayed significantly increased resistance to *P. falciparum* infection and DENV infection (Fig. 3). The inhibition of *P. falciparum* infection was even greater when *Csp_P* was introduced through the gametocyte-containing blood meal at 10^6^ CFU/ml (Fig. 3B), an effect most likely attributable to the larger number of ingested bacteria. *Csp_P* may inhibit pathogen infection directly through physical interaction with the pathogens or the production of anti-pathogen molecules. Alternatively, *Csp_P* may indirectly inhibit *Plasmodium* or dengue by (a) altering the long-term physiology or health of the mosquito such that pathogen infection is inhibited, (b) triggering a mosquito anti-pathogen response or (c) selecting for individuals that are more fit to resist *Csp_P* as well as DENV and *Plasmodium* infection. However, *Csp_P's in vitro* anti-pathogen activity (discussed below) suggests it has the potential to directly inhibit pathogen survival in the mosquito gut. Further studies are necessary to elucidate the mechanism by which *Csp_P* inhibits the pathogens in *vitro* and *in vivo*.

### *Csp_P* induces mosquito innate immune system genes

**Figure 4:**
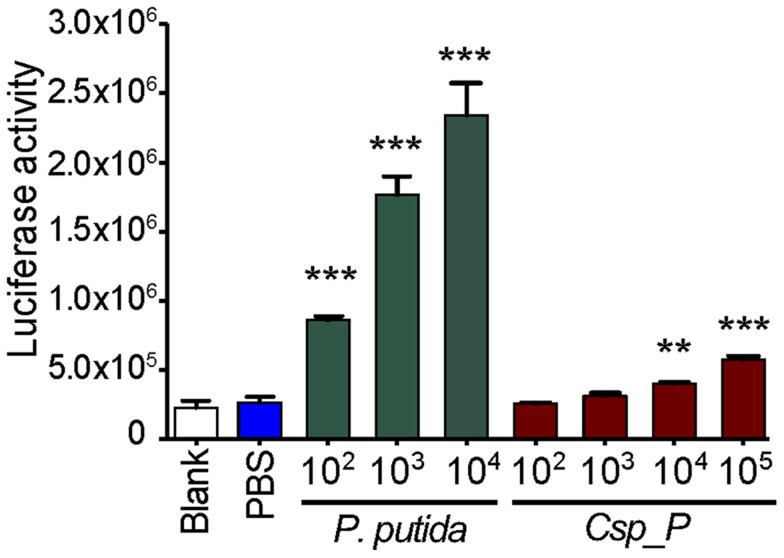
*Csp_P* elicits immune gene expression in the mosquito. Induction of the Cec1 promoter in the SUA-5B cell-line exposed to *P. putida* and *Chromobacterium sp. Csp_P*. SUA-5B cells expressing a luciferase reporter gene driven by a Cec1 promoter were exposed to increasing concentrations of *Csp_P* and *P. putida* bacteria. Differences between bacteria-treated samples and PBS control samples were assessed by Dunnett's Multiple Comparison Test (**, p<0.01; ***, p<0.001).

We have previously shown that the *An. gambiae* and *Ae. aegypti* midgut microbiota elicit basal immune activity by elevating the expression of several immune factors, including antimicrobial peptides and antipathogen factors [5], [7], [8], [23], [24]. To determine *Csp_P*'s potency in inducing the mosquito's innate immune system, we exposed mosquito SUA-5B cells to various concentrations of *Csp_P* and assayed for changes in the activity of a Cecropin1 promoter driving the expression of a luciferase reporter gene. We exposed these same cells to *Pseudomonas putida*, a Gram-negative bacterium that belongs to a bacterial genus commonly found in mosquito midguts [25]–[27]. This experiment showed that Cec1 expression increased with increasing *Csp_P* exposure, providing evidence that *Csp_P* is a potent immune elicitor (Fig. 4). We also compared the transcript abundance of mosquito immune genes in midguts from antibiotic-treated naïve mosqutioes to those from mosquitoes that had been provided a sugar source spiked with *Csp_P* (10^8^ CFU/ml for *An. gambiae* and 10^6^ CFU/ml for *Ae. aegypti*) 2 days earlier. We chose to assay gene expression at 2 days post exposure because this is the time at which increased mortality due to infection begins to occur. We hypothesized that infection levels and therefore any potential immune response would be high at this time. In *Ae. aegypti*, we found that cecropin E and G and defensin C displayed at least a 2-fold increase in transcript abundance in the midgut of *Ae. aegypti* colonized with *Csp_P* bacteria when compared to naïve controls (Fig. S1A). In *An. gambiae*, we found non-significant trends toward increased transcript abundance of the Rel2, FBN9 and cecropin genes and toward decreased transcript abundance of the defensin gene in the midgut tissue (Fig. S1B). These data represent a single time point post-infection, and while it is possible that additional time points may reveal dynamic patterns of *Csp_P*-induced changes in gene expression, our results generally agree with the cell culture data, and as a whole show that *Csp_P* has an immune-eliciting capacity in the mosquito gut.

### *Csp_P* inhibits *Plasmodium* development and abolishes dengue virus infectivity *in vitro*, independent of the mosquito

To test whether *Csp_P* could exert a direct anti-*Plasmodium* or anti-dengue effect *in vitro* that is independent of the mosquito, we performed experiments in which parasite development and virus infectivity were assayed after exposure to various preparations of either planktonic or biofilm cultures of *Csp_P*. Planktonic-state *Csp_P* was obtained by culturing *Csp_P* in liquid LB at 30°C overnight on a platform shaker. Biofilm was produced by culturing *Csp_P* in LB without agitation in a polystyrene 24-well plate at room temperature for 48 h, unless otherwise indicated. The anti-dengue and anti-*Plasmodium* activity of the following five different preparations of *Csp_P* was then tested: (a) 1 ml (10^8^ CFU/ml) planktonic-state liquid culture, (b) 1 ml (10^9^ CFU/ml) biofilm supernatant consisting of liquid LB drawn off freshly cultured biofilm, (c) 5 mg (10^9^ CFU/ml) fresh biofilm resuspended in 1× PBS, (d) 5 mg dessicated biofilm prepared from biofilm collected 1–2 days prior to assay and allowed to completely dessicate at room temperature and then rehydrated in 1× PBS, (e) 5 mg heat-inactivated biofilm prepared by heating biofilm at 90°C for 24 h, collected 1 day prior to assay.

**Figure 5:**
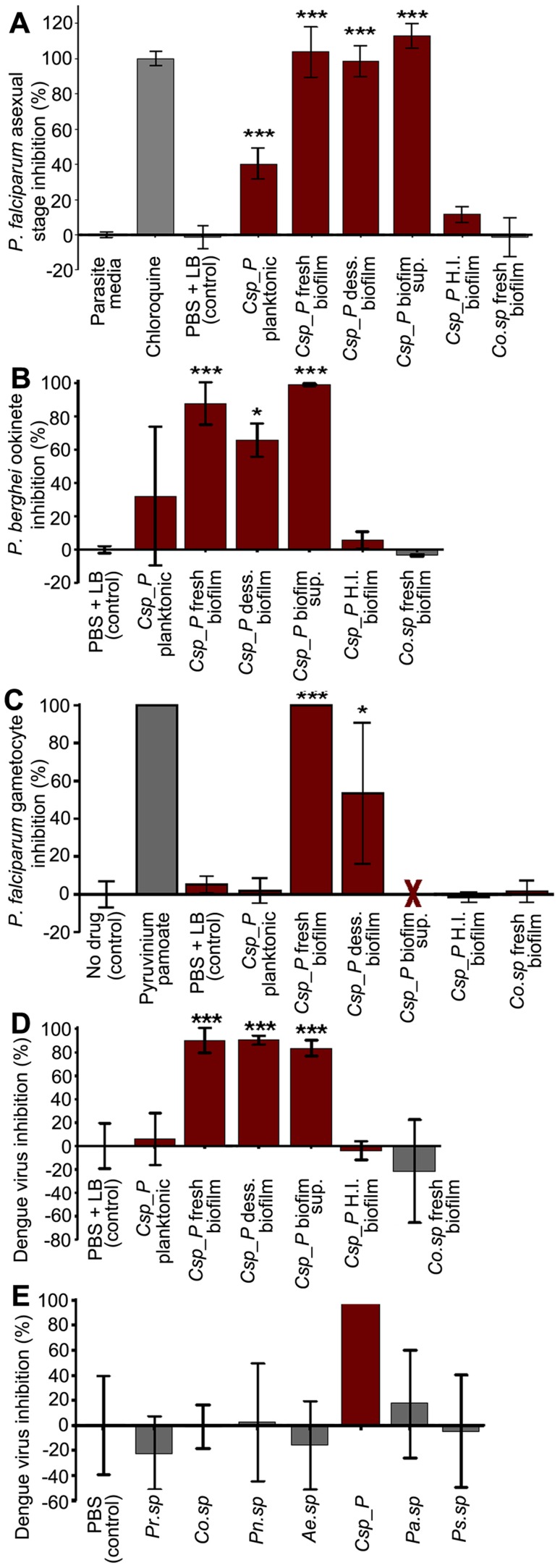
*Csp_P* has anti-*Plasmodium* and anti-dengue activity *in vitro*. *Csp_P* was grown under planktonic and/or biofilm conditions and tested for anti-pathogen activity independent of the mosquito. Five different preparations of *Csp_P* were tested: (a) planktonic-state liquid culture, (b) biofilm supernatant, (c) fresh biofilm, (d) dessicated biofilm, and (e) heat-inactivated biofilm. A fresh *Comamonas sp* biofilm was also tested as control. (A) *Csp_P* 36-h biofilm has anti-parasite activity against asexual-stage *P. falciparum.* *Csp_P* cultures were filtered using a 0.2-µm filter and mixed with ring-stage *P. falciparum* parasite cultures. SYBR green I was then added to each sample, and inhibition of asexual-stage *P. falciparum* by *Csp_P* was measured by assaying fluorescence relative to the negative control (parasite medium, standardized to 0% inhibition). Chloroquine was used as a positive control and standardized to 100% inhibition. We performed a Tukey's test on the raw data to determine whether each bacterial treatment differed significantly from the PBS+LB control (*** p<0.001). (B) *Csp_P* has anti-parasite activity against ookinete-stage *P. falciparum*. *Csp_P* bacterial preparations were filtered using a 0.2-µm filter and mixed with blood taken from female Swiss Webster mice infected with *Renilla* luciferase-expressing transgenic *P. berghei*. Ookinete-stage *P. berghei* parasite counts were determined using the *Renilla* luciferase assay system, and percent inhibition by *Csp_P* was calculated relative to the negative control (PBS+LB control, standardized to 0% inhibition). We performed a Tukey's test to determine whether each bacterial treatment differed significantly from the control (*p<0.05, ***, p<0.001). (C) *Csp_P* 42-h biofilm has anti-parasite activity against gametocyte-stage *P. falciparum*. *Csp_P* cultures were filtered using a 0.2-µm filter and mixed with gametocyte-stage *P. falciparum* cultures. Erythrocytes were examined for gametocytes using Giemsa-stained blood films collected 3 days after *Csp_P* exposure. The red X indicates that the supernatant caused hemolysis and was therefore unusable. We determined gametocyte density per 1000 RBCs for each sample and performed a Tukey's test to determine whether each bacterial treatment significantly differed from the PBS+LB control (*p<0.05, *** p<0.001). (D) *Csp_P* has anti-dengue activity. Each *Csp_P* bacterial preparation (75 µl, unfiltered) was mixed with 75 µl MEM containing dengue virus serotype 2 and incubated at room temperature for 45 min. Samples were then filtered through a 0.2-µm filter and used to infect BHK21-15 cells. Percent inhibition was calculated as the percent decrease in PFU/ml relative to the negative control (PBS+LB, standardized to 0% inhibition). We analyzed the significance of pairwise comparisons between each treatment and the control using a Tukey's test (***, p<0.001). (E) *Csp_P* has anti-dengue activity when virus is suspended in human blood. Biofilms from multiple bacteria were tested for anti-dengue activity. All bacteria tested were isolated from field-caught *Ae. aegypti* mosquitoes. *Ps.sp* = *Pseudomonas sp.*, *Pr.sp* = *Proteus sp.*, *Pn.sp* = *Paenobacillus sp.*, *Co.sp* = *Comamonas sp.*, *Pa.sp* = *Pantoea sp.*, *Ae.sp = Aeromonas sp.* [5]. The biofilm from each species was grown for 48 h at room temperature, and dengue virus mixed 1∶1 with human blood was added directly to the biofilm. After a 45-min incubation, the virus+blood/bilofilm solution was filtered and used to infect C6/36 cells. Biofilm sup = biofilm supernatant, H. I. biofilm = heat inactivated biofilm, dess. biofilm = dessicated biofilm re-suspended in 1× PBS.

Our *in vitro* assays showed that *Csp_P* exerts potent anti-*Plasmodium* activity against both asexual and sexual parasite stages. We exposed *P. falciparum* 3D7 asexual stage parasites to all five bacterial preparations *in vitro*. Because bacterial growth can interfere with determining parasite number, we removed bacterial cells by filtering all preparations though a 0.2-µm filter. We found that all filtrates from 36-h biofilm preparations (fresh, supernatant, and dessicated) possessed strong anti-*Plasmodium* activity, resulting in inhibition of asexual stage parasites at a level comparable to the chloroquine-treated positive control (p<0.001, Fig. 5A). We also detected moderate anti-asexual stage activity in planktonic *Csp_P* preparations (p<0.001), while heat-inactivated *Csp_P* biofilm and biofilm from another bacterial species, *Comamonas sp.*, had no inhibitory effect. We exposed an *in vitro Plasmodium* ookinete culture to all five filtered bacterial preparations to assess sexual-stage inhibition and found that the *Csp_P* 48-h biofilm (fresh, p<0.001; and dessicated, p<0.05) and biofilm supernatant (p<0.001) strongly blocked ookinete development (Fig. 5B). Exposure of the ookinete culture to the filtered planktonic *Csp_P* liquid culture resulted in a moderate but non-significant inhibition of ookinete development, and exposure to heat-inactivated *Csp_P* biofilm or *Comomonas sp.* biofilm filtrate had no effect on ookinete development (Fig. 5B). We also tested the effect of *Csp_P* bacterial preparations on *P. falciparum* gametocyte viability. Exposure to 42-h fresh biofilm filtrate resulted in 100% inhibition (p<0.001, Fig 5C) and exposure to 42-h dessicated biofilm resulted in approximately 60% inhibition (p<0.05, Fig. 5C) of *P. falciparum* gametocyte development. Gametocytemia could not be estimated for 42-h biofilm supernatant because this preparation caused hemolysis of RBCs (Fig. 5C). However, 36-h biofilm supernatant (which is not hemolytic) caused approximately 60% gametocyte inhibition when compared to the LB+PBS control (p = 0.06, Fig. S2).

To test the inhibitory effect of *Csp_P* preparations on dengue virus infectivity *in vitro*, we mixed dengue virus (10^6^ PFU/ml) in MEM 1∶1 with each of the five bacterial preparations of *Csp_P* for 45 min. Samples remained unfiltered during intial exposure to dengue and were filtered through a 0.2-µm filter before proceeding with standard plaque assays to avoid contamination of host cells. We found that exposure of dengue virus to a planktonic *Csp_P* culture did not affect its infectivity in BHK21-15 cells, whereas exposure to *Csp_P* biofilm, dessicated biofilm, or biofilm supernatant did abolish dengue virus infectivity (p<0.001, Fig. 5D). To better replicate the effect that *Csp_P* biofilm might have on dengue virus in human blood, we exposed dengue virus in human blood to *Csp_P* fresh biofilm for 45 min. We then filtered the blood+virus/biofilm mixture and assessed dengue virus infectivity by standard plaque assay. We found that fresh *Csp_P* biofilm displayed strong anti-dengue activity when the virus was suspended in human blood (Fig. 5E). *Csp_P* fresh biofilm was unique in its anti-dengue activity, since the biofilms of several other bacterial isolates from the guts of field-caught mosquitoes [5] did not exert any antiviral activity against dengue virus in human blood (Fig. 5E). The anti-dengue activity of *Csp_P* was apparently dependent on biofilm maturation, since biofilm grown for 24 h showed only weak inhibition when compared to 48-h biofilm (Fig. S3A). The *Csp_P* biofilm-associated anti-*Plasmodium* and antiviral activity was also heat-sensitive, since it could be inactivated through a 24-h incubation at 90°C (Fig. 5A–D).

Bacterial biofilms are composed of a matrix of extracellular polymeric substances containing polysaccharides, proteins, DNA, and secondary metabolites [12]. To investigate whether the anti-viral activity could simply be a result of virus particle sequestration by the biofilm, we mixed a dengue virus suspension with biofilm and incubated the mixture for a period of 45 min. Samples were then centrifuged, and viral RNA in the supernatants was quantified by RT-qPCR and compared between experimental (biofilm+DENV) and control (LB+DENV) treatments. Our results indicated that the dengue virus was not sequestered by *Csp_P* biofilm, since similar viral RNA copies were detected in the biofilm-exposed sample and the LB control sample (Fig. S3B). To investigate whether the loss of dengue virus infectivity was due to a biofilm-mediated change in the pH of the medium, we measured the pH of a dengue virus-*Csp_P* biofilm mixture at the end of a 45-min incubation period. The pH measurements showed an increase in the pH of the medium from 7.6 to 8.3 (Fig. S4A). A similar change in the pH was observed when we used the biofilms of other bacteria (*Pantoea sp.* Pasp_P and *Proteus sp.* Prsp_P) that do not affect dengue virus infectivity (Fig. S4A). To further investigate the effect of pH on dengue virus infectivity, we adjusted the pH of the MEM medium with NaOH and HCl to pH values of 5.0, 7.7, 8.5, and 10.0 prior to a 45-min incubation with the dengue virus. A decrease in virus infectivity was only observed after exposure to a pH of 5.0, suggesting that the moderate increase in pH did not mediate the *Csp_P* biofilm's inhibition of virus infectivity (Fig. S4B). We also showed that *Csp_P* biofilm does not exert a cytotoxic effect on insect or mammalian cells, as assessed by standard trypan blue cell staining (Invitrogen) (Fig. S5). We finally tested whether the *Csp_P* biofilm could influence the host cells' susceptibility to dengue virus infection by exposing C6/36 cells to *Csp_P* biofilm, then removing the biofilm through washes with PBS prior to dengue virus infection assays. This treatement did not influence the virus's ability to infect the host cells (Fig. S6), suggesting that the anti-DENV activity of *Csp_P* biofilm is not due to a reduced host cell susceptibility to the virus but is likely a direct anti-viral effect.

### *Csp_P* produces broad-spectrum antibacterial activity(ies)

**Figure 6:**
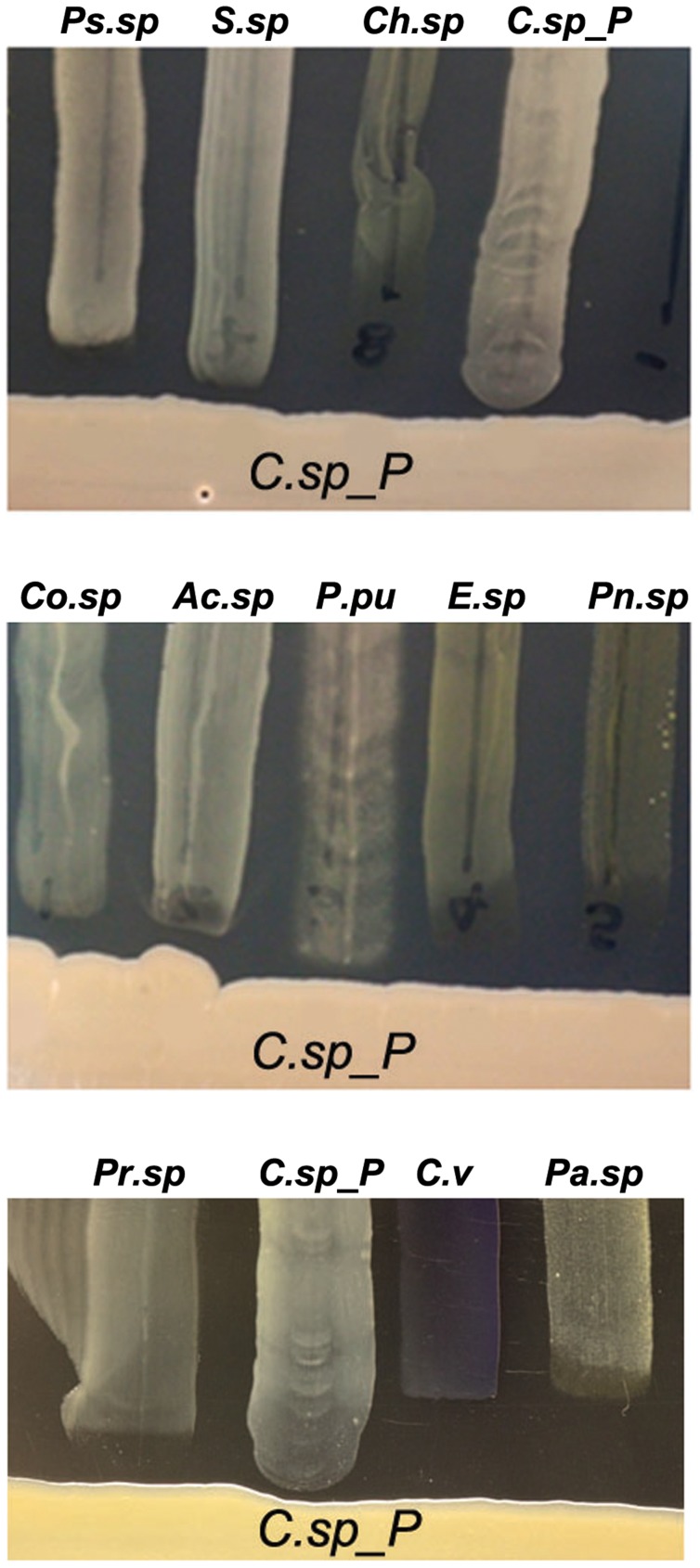
*Csp_P* has anti-bacterial activity against many species commonly found in the midguts of *Aedes* and *Anopheles* mosquitoes. *Csp_P* was streaked on LB agar along with multiple bacterial species, and plates were observed for formation of zones of inhibition around *Csp_P. Ps.sp* = *Presudomonas sp.*, *Pr.sp* = *Proteus sp.*, *C.sp_P* = *Chromobacterium sp_P*, *C.v* = *C. violaceum*, *Pn.sp* = *Paenobacillus sp.*, *Co.sp* = *Comamonas sp.*, *Ac.sp* = *Acinetobacter sp.*, *P.pu* = *Pseudomonas putida*, *E.sp* = *Enterobacter sp.*, *Pa.sp* = *Pantoea sp.*, *S.sp = Serratia sp.*, *Ch.sp = Chryseobacterium sp.* [3], [5], [7].

To provide baseline information on the potential production of antibacterial factors by *Csp_P*, we performed a basic growth inhibition assay by investigating the ability of a number of other mosquito midgut-derived bacterial isolates (*Ae. aegypti*-derived microbiota: *Ps.sp* = *Pseudomonas sp.*, *Pr.sp* = *Proteus sp., C.sp_P* = *Chromobacterium sp_P*, *C.viol* = *C. violaceum*, *Pn.sp* = *Paenobacillus sp.*; *An. gambiae*-derived microbiota: *Co.sp* = *Comamonas sp.*, *Ac.sp* = *Acinetobacter sp.*, *P.pu* = *Pseudomonas putida*, *E.sp* = *Enterobacter sp.*, *Pa.sp* = *Pantoea sp., Ps.sp = Pseudomonas sp., S.sp = Serratia sp., Ch.sp = Chryseobacterium sp.*) to grow in proximity to *Csp_P* on LB agar plates (Fig. 6). *Csp_P* was streaked on LB agar and allowed to grow for 48 hours. Midgut-derived bacterial isolates were then vertically streaked up to the Csp_P streak, and allowed to grow in the presence of *Csp_P*. This assay showed a prominent growth inhibition zone around the *Csp_P* streak, with inhibition of the growth of all the bacterial isolates that were derived from field-collected *Ae. aegypti* [5] and *An. gambiae* [3], including a close relative known for its production of a variety of bioactive factors, *C. violaceum* (Fig. 6A).

### Conclusions

Insect-bacteria associations can influence vector competence in multiple ways; these include shortening the insect's life span, blocking infection with human pathogens by the production of bioactive anti-pathogen factors, and eliciting the insect immune system. We have identified a *Chromobacterium sp.* (*Csp_P*) bacterium from the midgut of field-derived *Aedes aegypti* that exerts broad-spectrum anti-pathogen activity against *Plasmodium* and dengue virus. Specifically, *Csp_P* renders *An. gambiae* and *Ae. aegypti* more resistant to infection by the human malaria parasite *Plasmodium falciparum* and dengue virus, respectively. *Csp_P* inhibits the growth of a variety of other bacterial species found in the mosquito midgut and is capable of rapidly colonizing the mosquito midgut. *Csp_P* appears to exert entomopathogenic activity, since exposure of larvae to *Csp_P* in the breeding water and ingestion of *Csp_P* by adult mosquitoes result in high mosquito mortality. It is possible that *Csp_P* could be effectively used as a transmission blocking agent if it was delivered to mosquitoes through baited sugar traps [28]. *Csp_P*'s ability to colonize the mosquito gut could be further enhanced through established selection procedures based on consecutive passages of the bacterium through the mosquito intestine [29]. *Csp_P* could be used alone in baited sugar traps or in combination with other microbes that have also been shown to either kill the mosquito or reduce pathogen infection, or both, when present in the mosquito gut [3], [24]. The larvicidal activity of *Csp_P* also renders it interesting for potential use in mosquito population suppression. The anti-pathogen activities of *Csp_P* appear to be mediated by bacteria-produced metabolites that also inhibit parasite and virus infection *in vitro*, making them interesting as possible lead compounds for transmission blocking and therapeutic drug development. The entomopathogenic, anti-bacterial, anti-viral, and anti-*Plasmodium* properties of *Csp_P* make this bacterium a particularly interesting candidate for the development of novel control strategies for the two most important vector-borne diseases, and they therefore warrant further in-depth study.

## Methods

### Ethics statement

This study was carried out in strict accordance with the recommendations in the Guide for the Care and Use of Laboratory Animals of the National Institutes of Health. Mice were only used for mosquito rearing as a blood source according to approved protocol. The protocol was approved by the Animal Care and Use Committee of the Johns Hopkins University (Permit Number: M006H300). Commercial anonymous human blood, supplied from Interstate Blood Bank Inc., was used for *Plasmodium* and dengue virus infection assays in mosquitoes, and informed consent was therefore not applicable. The Johns Hopkins School of Public Health Ethics Committee has approved this protocol. Mosquito collections were performed in residences after owners/residents permission.

### Mosquito rearing and antibiotic treatment

*Aedes aegypti* mosquitoes were from the Rockefeller strain, and *Anopheles gambiae* mosquitoes were from the Keele strain. Both were maintained on a 10% sugar solution at 27°C and 95% humidity with a 12-h light/dark cycle. Sterile cotton, filter paper, and sterilized nets were used to maintain the cages as sterilely as possible. For experiments utilizing aseptic mosquitoes, females were maintained on a 10% sucrose solution with 20 U penicillin and 20 µg streptomycin from the first day post-eclosion until 1–2 days prior to challenge. The effectiveness of the antimicrobial treatment was confirmed by colony forming unit assays prior to blood-feeding or bacterial challenge.

### Introduction of bacteria via sugar meal

In cases where mosquitoes were antibiotic treated, reintroduction of bacteria through a sugar meal was done by first treating mosquitoes with antibiotics for 2–3 days after emergence, then providing them with 10% sucrose (for *An. gambiae*) or sterile water (for *Ae. aegypti*) for 24 h post-antibiotic treatment. When mosquitoes were not antibiotic treated, they were maintained on 10% sucrose for 2–5 days post emergence. *Ae. aegypti* were given sterile water during the final 24 hours of this period. In all cases, mosquitoes were then starved overnight and fed for 24 h on cotton strips moistened with a 1.5% sucrose solution containing *Csp_P* at a final concentration of approximately 10^8^ CFU/ml for *An. gambiae* and 10^6^ CFU/ml for *Ae. aegypti*. In some experiments (Figures 1 and 2), *Ae. aegypti* mosquitoes were also fed *Csp_P* at a final concentration of 10^10^ CFU/ml.

### Assaying prevalence and bacterial load of *Csp_P*

In antibiotic treated mosquitoes, midguts were dissected three days post ingestion of *Csp_P*, homogenized in 1× PBS and plated on LB agar. Colonies were then counted to estimate colony forming units (CFUs) per midgut as well as prevalence of *Csp_P*. In mosquitoes not treated with antibiotics, prevalence and/or bacterial load was estimated in one of two ways. For *An. gambiae*, midguts were dissected at one and two days post *Csp_P* ingestion, homogenized in 1× PBS and serial dilutions of the homogenate were plated on LB agar supplemented with ampicillin (10,000 ug/ml). *Csp_P* is highly resistant to ampicillin and grows readily even at this high concentration. We verified that *Csp_P* was the only bacterium growing on antibiotic treated plates by first confirming that all colonies that grew were similar in color, growth rate and colony morphology. 16s rDNA was then sequenced from a subset of colonies and verified to match the sequence of *Csp_P* from pure freezer stock.

It was not possible to use this method for *Ae. aegypti* because their midguts commonly contained other highly ampicillin-resistant bacteria. These contaminants grew to very high numbers on the ampicillin-treated plates and interfered with the detection of *Csp_P*. DNA was therefore extracted using the ZR Soil Microbe DNA MicroPrep kit (Zymo Research) from samples dissected 1 and 3 days after feeding on a sugar meal containing either PBS or *Csp_P* (10^10^ CFU/ml).The manufacturer's protocol was altered in the following way: instead of using lysis buffer to disrupt cells, each midgut was put in 500 µl 1× PBS, 25 µl lysozyme (10 mg/ml) and 7.5 µl mutanolysin (10 KU/ml) were added and the samples were incubated at 37°C for 1.5 h. 15 µl proteinase K and 25 µl 10% SDS were then added, samples were incubated at 55°C for 1 h, and the standard protocol was then resumed. A diagnostic PCR was performed to assess the presence of *Csp_P* in each individual midgut. Primers were designed to amplify a 415 bp fragment of the *Csp_P* hydrogen cyanide synthase B gene and the primers were verified to be *Csp_P*-specific using Primer BLAST from NCBI (Forward primer: 5′AGGGCGTAACCCTGGACTAT 3′, Reverse primer: 5′ CCGAAGGAACTGGCTTCGTA 3′). PCR was performed with the above primers using 10 ng DNA as template and Phusion High-Fidelity DNA Polymerase according to the manufacturer's instructions, with the following exceptions: 0.5 µl of each primer (10 µm) was used, and 0.25 µl BSA was added to each reaction. Cycling conditions were as follows: 95°C for 30 seconds, [95°C for 30 s, 65°C for 30 s, 72°C for 45 s]×27 cycles, 72°C for 10 minutes. 8 µl of each sample was run on a 1% agarose gel and visualized at 400 ms exposure. A visible 415 bp band was considered positive evidence of *Csp_P* bacteria (see Fig. S7 for a representative example). A very faint band was detected in one of 40 PBS samples, suggesting a minor contamination event or the presence of another bacterium with high sequence identity to *Csp_P*. This was an isolated incident and was not seen in any other PBS samples. Two independent PCR products were sequenced from *Csp_P* fed samples and verified to be a perfect match to the sequence obtained from *Csp_P* sequenced directly from freezer stock. To serve as a positive control and to allow estimation of the sensitivity of the diagnostic PCR, a standard curve was run in which a range of 10^7^–10^1^ copies of the *Csp_P* hcn B PCR product was used as template. In this way, it was possible to estimate the minimum detection threshold of this assay. Using the above mentioned PCR conditions, a band was detectable in wells containing 10^3^ initial copies of the hcn B product but not in wells containing 10^2^ initial copies, suggesting that this assay is capable of detecting a minimum of 10^3^ copies of *Csp_P*/midgut.

### Introduction of bacteria via blood meal

At 2 days prior to blood feeding, sucrose was removed, and the mosquitoes were given sterile water. They were then starved for 12 h prior to blood feeding. *Csp_P* was grown overnight in liquid LB at 30°C. The overnight culture (1 ml) was then pelleted, washed with 1× PBS, and resuspended in 1× PBS to OD_600_ = 1.0, which equals a concentration of approximately 10^8^ CFU/ml. Mosquitoes were then allowed to membrane-feed on blood containing bacteria or 1× PBS as a control (blood mixture: 50% 1.0 OD_600_ bacterial culture or 1× PBS, 40% blood, 10% human serum). Bacteria-fed adult females ingested approximately 10^5^ CFU per mosquito.

### Exposure of larvae to *Csp_P*

At 2–4 days post-hatching, larvae were placed in cell culture plates in groups of 10 per well. Each well contained 5 ml sterile water plus a small amount of larval food (liver powder, tropical fish flake food, and rabbit food pellets mixed in a 2∶1∶1 ratio). We then added 50 µl of an overnight culture of *Csp_P* diluted to OD_600_ = 1.0 (10^8^ CFU/ml) to each well; 1× PBS was added to control wells, and mortality was monitored in all wells for a 5-day period.

### Cell culture maintenance, mosquito infections with dengue virus, and titration of infected midguts

Dengue virus serotype 2 (New Guinea C strain, DENV-2) was propagated in the C6/36 mosquito cell line according to previously published methods [8]. In brief, cell line infection was allowed to proceed for 5–7 days, at which time the cells were harvested with a cell scraper and lysed by freezing and thawing in dry CO_2_ and a 37°C water bath, then centrifuged at 800 g for 10 min. Dengue virus serotype 2 was isolated and mixed 1∶1 with commercial human blood and used for infections as described in [8]. Mosquitoes that had previously fed on *Csp_P* bacteria-sucrose solution were starved overnight prior to dengue virus infection. Infected mosquitoes were collected at 7 days post-infection and surface-sterilized by dipping them in 70% ethanol for 1 min and then rinsing them twice in 1× PBS for 2 min each. Midgut dissection was done in one drop of 1× PBS under sterile conditions, and the midgut was transferred to a microcentrifuge tube containing 150 µl of MEM. Midguts were homogenized using a Kontes pellet pestle motor, filtered, and stored at −80°C until ready for virus titration.

Dengue virus titration of infected midguts was done as previously reported [8], [30]. In brief, the infected midgut homogenates were serially diluted and inoculated into C6/36 cells in 24-well plates. After an incubation of 5 days at 32°C and 5% CO_2_, the plates were fixed with 50%/50% methanol/acetone, and plaques were assayed by peroxidase immunostaining using mouse hyperimmune ascitic fluid specific for DENV-2 as the primary antibody and a goat anti-mouse HRP conjugate as the secondary antibody. In addition, where indicated, dengue virus plaque assays were conducted in BHK-21 cells. At 5 days post-infection, the 24-well plates were fixed and stained with crystal violet. Plaques (formed by cells with cytopathic effect) were counted and analyzed.

### *P. falciparum* cultivation, mosquito infections, and oocyst counts

*P. falciparum* strain NF54 was maintained in continuous culture according to the method described by Tragger and Jensen [31]. In brief, *P. falciparum* was grown in O+ red blood cells (RBCs) at 2% hematocrit and RPMI 1640 medium supplemented with glutamine, HEPES, hypoxanthine, and 10% O+ human serum. To maintain a microaerophilic environment, parasites were maintained in a candle jar at 37°C. Use of human erythrocytes to support the growth of *P. falciparum* was approved by the internal review board of the Bloomberg School of Public Health. Gametocytemia and exflagellation events were assessed after 18 days of *P. falciparum* culture. The gametocyte culture was centrifuged and diluted in a mixture of RBCs supplemented with serum. Mosquitoes were rendered aseptic via antibiotic treatment and then fed on membrane feeders for 30 min with blood containing *P. falciparum* gametocytes. *Csp_P* was either added directly to the infectious blood meal (bacterial concentration = 10^6^ CFU/mL) or introduced via sugar meal as described above 3 to 4 days prior to the infectious blood meal. On the same day as the blood meal, mosquitoes were sorted, and the unfed mosquitoes were removed. At 7 to 8 days after blood feeding, the fed mosquitoes were dissected, and their midguts were stained with 0.1% mercurochrome. The number of oocysts per midgut was determined with a light-contrast microscope, and the median was calculated for the control and each experimental condition. More than three independent replicates were used per group.

### *Csp_P* culture preparations for *in vitro* anti-*Plasmodium* and anti-dengue activity assays

To grow bacteria in planktonic conditions, we spiked 5 ml sterile LB with 5 µl of bacterial freezer stock and allowed the culture to grow overnight at 30°C with shaking. We then diluted planktonic cultures to OD_600_ = 1.0 (±0.1) with additional sterile LB broth which, for *Csp_P*, results in a concentration of approximately 10^8^ CFU/ml. To grow bacteria under biofilm conditions, we dispensed 1 ml of sterile LB into each well of a 24-well cell culture plate and spiked each well with 1 µl of bacterial freezer stock. We then allowed the culture to grow at room temperature without shaking for 48 h. *Csp_P* biofilm supernatant was harvested from single bacterial culture wells containing 48-h biofilm and was found to have an average bacterial concentration of approximately 10^9^ CFU/ml. To harvest fresh biofilm, we removed the supernatant from five wells containing 48-h biofilm, resuspended the biofilm from each well in 100 µl 1× PBS and pooled the five wells. For *Csp_P*, this pooled biofilm solution contained approximately 10^9^ CFU/ml and an average of 5 mg of biofilm (dry weight). To obtain desiccated biofilm, we collected the fresh biofilm from five wells as indicated, centrifuged the biofilm at 5000 rpm for 2.5 min, removed the PBS supernatant, and allowed the biofilm to dry at room temperature. On the day of the experiment, we resuspended the five wells of desiccated biofilm in 500 µl 1× PBS to mimic the fresh biofilm treatment. To heat-inactivate the fresh biofilm, we collected fresh biofilm as indicated and incubated samples at 90°C for 24 h prior to the experiment.

### *In vitro* anti-*Plasmodium* activity assays

We prepared *Csp_P* bacterial cultures as described above and filtered all samples through a 0.2-µm filter.

*Asexual-stage assay:* Inhibition of asexual-stage *P. falciparum* was assessed using a SYBR green I-based fluorescence assay as described earlier [32]. *Csp_P* biofilm was grown for 36 h for this experiment because 48-h biofilm causes hemolysis of RBCs (Fig. S8), which interferes with the assay. Parasites were synchronized using 5% sorbitol [33]; 5 µl of each bacterial preparation was dispensed in triplicate wells of 96-well microplates, followed by addition of 95 µl of synchronous ring-stage *P. falciparum* cultures at 1% hematocrit and 1% parasitemia. Chloroquine (250 nM) was used as a positive control, and parasite growth medium was used as a negative control. After 72 h of incubation in a candle jar at 37°C, an equal volume of SYBR green-I solution in lysis buffer (Tris [20 mM; pH 7.5], EDTA [5 mM], saponin [0.008%; w/v], and Triton X-100 [0.08%; v/v]) was added to each well and mixed gently, then incubated 1–2 h in the dark at room temperature. Plates were read on a fluorescence plate reader (HTS 7000, Perkin Elmer), with excitation and emission wavelengths of 485 and 535 nm, respectively. Percent inhibition was calculated relative to negative (0% inhibition) and positive controls (100% inhibition). Three biological replicates were assayed.

*Ookinete-stage assay:* To assess inhibition of ookinete-stage *P. berghei* parasites, female Swiss Webster mice (6–8 weeks old) were infected with a transgenic strain of *P. berghei* that expresses *Renilla* luciferase. Starting at 3 days post-infection, exflagellation assays were performed until at least 20 exflagellation events were recorded in a 20× field. At this time, mice were bled by heart puncture using a heparinized needle, and the blood was diluted in 10 volumes of ookinete medium (RPMI 1640, 10% FBS, 50 mg/ml hypoxanthine, and 2 mg/ml NaHCO^3^, pH 8.3) with 4% mouse RBC lysate. Samples (50 µl) of each bacterial preparation were then mixed with the infected blood and incubated for 24 h at 19°C. Ookinete counts were determined using the *Renilla* luciferase assay system (Promega, USA) according to the manufacturer's instructions. The experiment was performed on two independent days, and each sample was assayed in triplicate on each day.

*Gametocyte-stage assay*: Inhibition of gametocyte-stage *P. falciparum* by *Csp_P* was assessed as described previously [34]. To prevent hemolysis of RBCs, *Csp_P* biofilm was grown for 36 and 42 hours for this experiment. In brief, NF54 *P. falciparum* cultures were started at 0.5% asexual parasitemia and 4% hematocrit. *Csp_P* bacterial preparations were added 15 days after *Plasmodium* cultures were initiated, and gametocytemia was determined 18 days after culture initiation. At least three biological replicates were tested for each culture preparation. More than 500 erythrocytes were examined for gametocytes across Giemsa-stained blood films from each sample.

### *In vitro* anti-dengue activity assays

We prepared *Csp_P* bacterial cultures as described above (planktonic state, biofilm, biofilm supernatant, dessicated biofilm, and heat-inactivated biofilm), mixed 75 µl of each bacterial culture preparation with 75 µl of MEM containing dengue virus serotype 2 and incubated the mixture at room temperature for 45 min. Samples were then filtered through a 0.2-µm filter, serially diluted, and used to infect BHK21-15 cells. Plaque assays were conducted as described above to assess dengue virus infectivity. Percent inhibition was calculated as the percent decrease in PFU/ml relative to the PBS+LB control, which was standardized to 0% inhibition. The experiment was performed on two independent days, and each assay was performed in triplicate on each day. In experiments in which dengue was mixed with human blood before exposure to *Csp_P*, bacterial biofilms were not removed from the cell culture plate. Rather, dengue virus was mixed 1∶1 with human blood, and 150 µl of this mixture was added directly to each well containing *Csp_P* biofilm and incubated for 45 min at 30°C. Following this incubation period, the blood-dengue virus solution was mixed with the biofilm, and 50 µl of the mixture was then drawn from the well, diluted in MEM, and filtered through a 0.2-µm filter. The resulting filtrate solution was then serially diluted and used to infect C6/36 cells.

### Assay for sequestration of viral particles by *Csp_P* biofilm

To assess whether the anti-dengue activity of *Csp_P* was due to sequestration of DENV by the *Csp_P* biofilm, we mixed a dengue virus suspension with *Csp_P* 48 hr biofilm or LB broth and incubated it for a period of 45 min. Samples were then centrifuged at 5,000 rpm for 5 min. The supernatants were collected, and RNA was extracted from equal volumes (50 µl) of experimental (biofilm+DENV) and control (LB+DENV) samples using the RNeasy kit (Qiagen). Comparison of viral RNA loads in the extracted supernatant was done via RT-qPCR relative quantification, using 2 µl of the viral RNA in a 20-µl reaction volume.

### Assessing pH effects on dengue virus infectivity

The pH of bacterial biofilms and supernatants was assessed with a micro-pH electrode (Lazar Lab) at room temperature. Effects of pH changes on dengue virus infectivity were assessed by adjusting the pH of the MEM with NaOH and HCl until the desired range of pH values was obtained: 5.0, 7.7, 8.5, and 10.0. The pH-adjusted MEM was then mixed with dengue virus-laden blood and incubated for 45 min prior to serial dilution and infection of C6/36 cells.

### Cell viability assays

Cell viability assays on the mosquito cell line C6/36 and the vertebrate cell line BHK-21 were performed via trypan blue staining (0.4%, Invitrogen) according to the manufacturer's instructions. In brief, 50 µl of suspended cells were placed in a microcentrifuge tube and mixed with 10 µl of *Csp_P* filtered fresh biofilm or PBS as a control. C6/36 cells were incubated at 32°C and BHK-21 cells were incubated at 37°C+5% CO_2_ for 45 min. Cells were then mixed with 12 µl of 0.4% trypan blue stain. The mixture was allowed to stand for 5 min at room temperature and then loaded into a hemocytometer for cell viability assessment and counting under a microscope.

### Assay of the effects of *Csp_P* biofilm on host cell susceptibility to DENV

To assess whether exposure to *Csp_P* biofilm changes the susceptibility of the host cell to DENV, we conducted assays exposing C6/36 cells to *Csp_P*-filtered biofilm prior to dengue virus infection. Cells were grown to 80% confluency; the cell medium was then removed, washed once with 1× PBS, and then overlaid with 100 µl of *Csp_P* biofilm that had been filtered using a 2-µm filter or with 1× PBS (control) for about 10 min. Plates were then washed three times with 1× PBS and then infected with 100 µl of dengue virus for about 45 min. Cells were assessed for plaque formation at 6 days post-infection.

### Hemolysis assay

Human erythrocytes were washed with RPMI 1640 medium until the supernatant was visually free of hemoglobin pigment. The washed erythrocytes were suspended in malaria complete medium to yield a 1% hematocrit. Filtered *Csp_P* biofilm was mixed with erythrocytes and incubated up to 24 h at 37°C. To separate lysed RBC cytosol from whole RBCs, the suspension was centrifuged at 2000 rpm for 5 min. The resulting supernatant was carefully aspirated and plated in new 96-well microplates. Control erythrocytes without any bacterial material were used as a negative control (blank), and freeze-thawed erythrocyte lysate was used as positive control (100% hemolysate). To determine the % lysis in test samples, plates were read at 405 nm in an ELISA plate reader (HTS 7000 Perkin Elmer), and the reading was expressed as a fraction of the positive control.

### Real-time qPCR assays

To conduct real-time PCR assays, RNA samples were treated with Turbo DNase (Ambion, Austin, Texas, United States) and reverse-transcribed using M-MLV Reverse Transcriptase (Promega, USA). The real-time PCR assays were performed using the SYBR Green PCR Master Mix Kit (Applied Biosystems, Foster City, California, USA) in a 20-µl reaction volume; all samples were tested in duplicate. The ribosomal protein S7 gene was used for normalization of cDNA templates. Primer sequences used in these assays are given in Table S1.

### Statistical analysis

The Mann-Whitney U test, one-way ANOVA with Dunnett's post-test and pairwise Log-Rank tests for survival analysis were conducted using the GraphPad Prism statistical software package (Prism 5.05; GraphPad Software, Inc., San Diego, CA). Data in Figure 5 were analyzed using an ANOVA, followed by a Tukey's test in R (R Foundation for Statistical Computing).

## Supporting Information

Data S1 **Raw data for** **figures 1** **–** **5** **.**
(XLSX)

Figure S1 ***Csp_P*** **elicits immune gene expression in the mosquito midgut.** Changes in the abundance of immune effector gene transcripts in the midgut of (A) *Ae. aegypti* and (B) *An. gambiae* mosquitoes were measured after the introduction of *Csp_P* via a sugar meal. For each gene, PBS controls were standardized to a value of 1.0, and *Csp_P*-induced changes in gene expression are shown as -fold change above or below PBS-fed controls. CecG = cecropin G, DefC = defensin C, LysC = lysozyme C, CecE = cecropin E, Cec1 = cecropin 1, Def1 = defensin 1, PGRP-LC = peptidoglycan recognition receptor LC, Rel2 = Relish-like NF-κB transcription factor 2, Tep1 = thioester protein 1, LRRD7 = leucine-rich repeat domain protein 7 (a.k.a. APL2 and LRIM17), FBN9 = fibronectin 9. Mann Whitney Tests comparing deltaCT values between bacteria-fed and PBS-fed mosquitoes for each gene were performed to determine significance (*, p<0.05).
(TIF)

Figure S2 **Effect of 36-h biofilm on gametocyte-stage** ***P. falciparum*** **.** *Csp_P* cultures were filtered using a 0.2-µm filter and mixed with gametocyte-stage *P. falciparum* cultures. Erythrocytes were examined for gametocytes using Giemsa-stained blood films collected 3 days after *Csp_P* exposure. We determined gametocyte density per 1000 RBCs for each sample and performed a Tukey's test to determine whether each bacterial treatment significantly differed from the PBS+LB control. No treatments were significant, but biofilm 36-h supernatant trended toward significance (p = 0.06)
(TIF)

Figure S3 **(A) Anti-dengue activity of fresh** ***Csp_P*** **biofilm is only weakly present after 24 h of growth at room temperature and becomes highly potent after 48 h of growth.** Dengue virus was mixed 1∶1 with human blood and directly exposed to *Csp_P* biofilm grown for 24 or 48 h. Samples were incubated for 45 min and then collected, filtered, and used to infect C6/36 cells. (B) Dengue virus particles are not sequestered by *Csp_P* biofilm. We mixed dengue virus with *Csp_P* biofilm and incubated the mixture for 45 min. We then centrifuged samples and used qRT-PCR to quantify viral RNA in the supernatant of the experimental (biofilm+DENV) and control (LB+DENV) treatments.
(TIF)

Figure S4 **(A) Assessing changes in pH caused by** ***Csp_P*** **biofilm.** We exposed dengue virus to *Csp_P* biofilm, incubated for 45 min, and measured the pH of the medium. (B) Assessing the effect of pH on dengue virus infectivity. We experimentally adjusted the pH of the MEM medium using NaOH and HCl to values of 5.0, 7.7, 8.5, and 10.0. We mixed the pH-adjusted media with dengue virus-laden human blood and incubated for 45 min., then collected and filtered the virus and used it to infect C6/36 cells.
(TIF)

Figure S5 **Crude biofilm extract does not have cytotoxic effects on insect or mammalian cells.** We used trypan blue staining (0.4%, Invitrogen) to assay cell viability of BHK21-15 cells (A) and C6/36 cells (B) after a 45 min exposure to filtered *Csp_P* fresh biofilm. Difference in cell viability due to *Csp_P* exposure were non-significant for both cell lines (Mann Whitney Test).
(TIF)

Figure S6 **Exposure to** ***Csp_P*** **biofilm does not alter the insect cells' susceptibility to dengue virus.** We filtered *Csp_P* biofilm using a 0.2-µm filter and exposed C6/36 cells (grown to 80% confluency) to the bacterial filtrate for 45 min. *Csp_P* biofilm filtrate was then washed from the cells using 1× PBS, and cells were infected with dengue virus. Cells were assessed for plaque formation at 6 days post-infection.
(TIF)

Figure S7 **Representative gels from PCR diagnostic to assay presence of** ***Csp_P*** **in** ***Aedes*** **mosquito midguts.** We used 10 ng of DNA from each *Aedes* female fed a sugar meal containing either PBS or *Csp_P* at a final concentration of 10^10^ CFU/ml. Using 10 ng of DNA from each sample as template, we performed a PCR using primers specific to the *Csp_P* hydrogen cyanide synthase B gene.
(TIF)

Figure S8 ***Csp_P*** **biofilm is hemolytic when exposed to human red blood cells.** We mixed filtered *Csp_P* fresh biofilm with human erythrocytes, incubated 24 h at 37°C and centrifuged at 2000 rpm for 5 min. We then removed the supernatant and assayed absorbance at 405 nm in an ELISA plate reader (HTS 7000 Perkin Elmer). 1× PBS was used as a negative control and saponin as a positive control.
(TIFF)

Table S1 **List of gene primers used in gene expression analyses of mosquito tissues post-bacterial challenge.**
(DOCX)
