# Supplementary figures and images for: Chromobacterium Csp_P Reduces Malaria and Dengue Infection in Vector Mosquitoes and Has Entomopathogenic and In Vitro Anti-pathogen Activities
Source: PLoS Pathog. 2014 Oct 23;10(10):e1004398. doi: 10.1371/journal.ppat.1004398 (PMC4207801; doi:10.1371/journal.ppat.1004398)

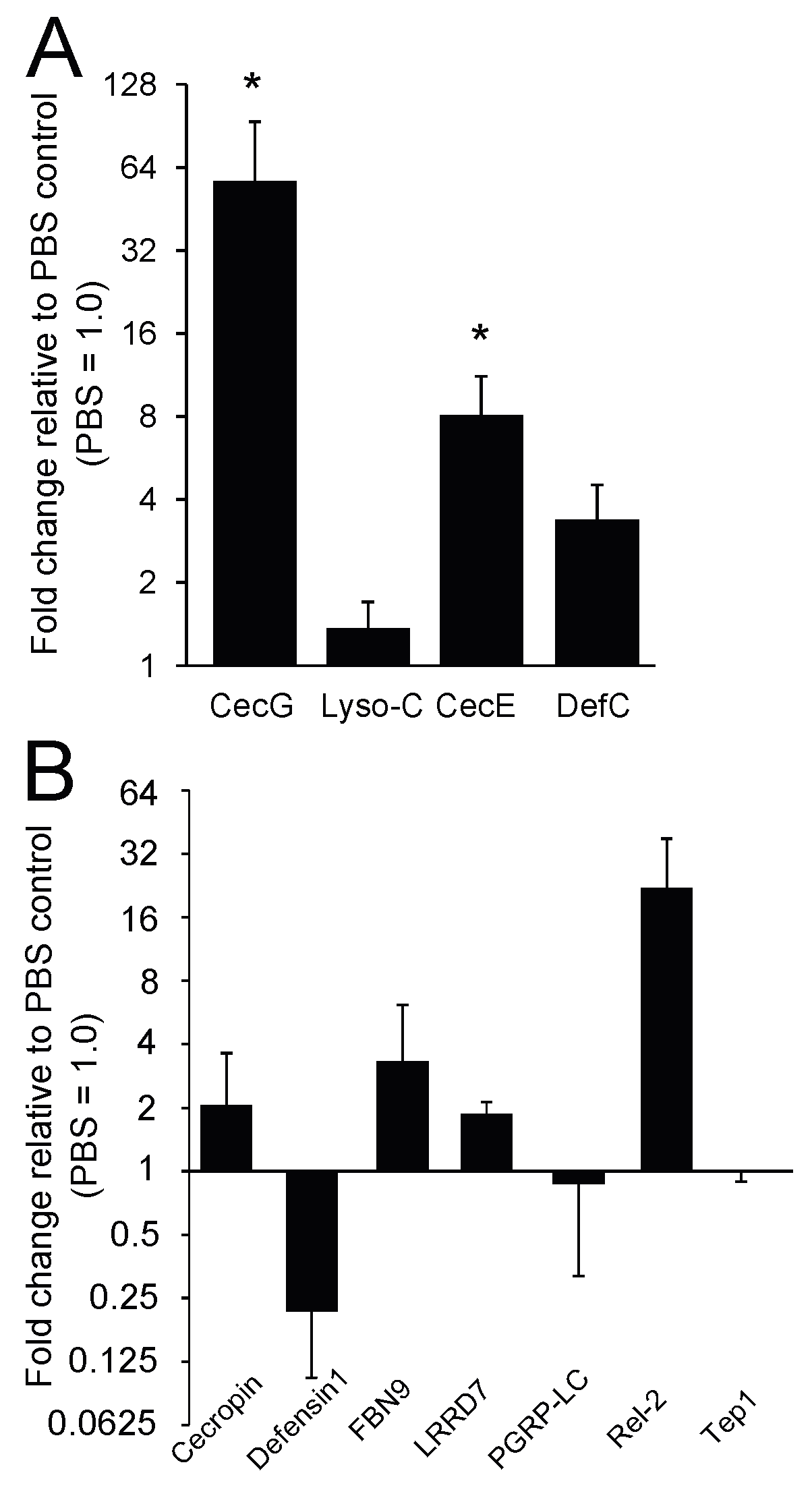

Supplement: Figure S1 — Csp_P elicits immune gene expression in the mosquito midgut. Changes in the abundance of immune effector gene transcripts in the midgut of (A) Ae. aegypti and (B) An. gambiae mosquitoes were measured after the introduction of Csp_P via a sugar meal. For each gene, PBS controls were standardized to a value of 1.0, and Csp_P-induced changes in gene expression are shown as -fold change above or below PBS-fed controls. CecG = cecropin G, DefC = defensin C, LysC = lysozyme C, CecE = cecropin E, Cec1 = cecropin 1, Def1 = defensin 1, PGRP-LC = peptidoglycan recognition receptor LC, Rel2 = Relish-like NF-κB transcription factor 2, Tep1 = thioester protein 1, LRRD7 = leucine-rich repeat domain protein 7 (a.k.a. APL2 and LRIM17), FBN9 = fibronectin 9. Mann Whitney Tests comparing deltaCT values between bacteria-fed and PBS-fed mosquitoes for each gene were performed to determine significance (*, p<0.05). (TIF) [file ppat.1004398.s002.tif]

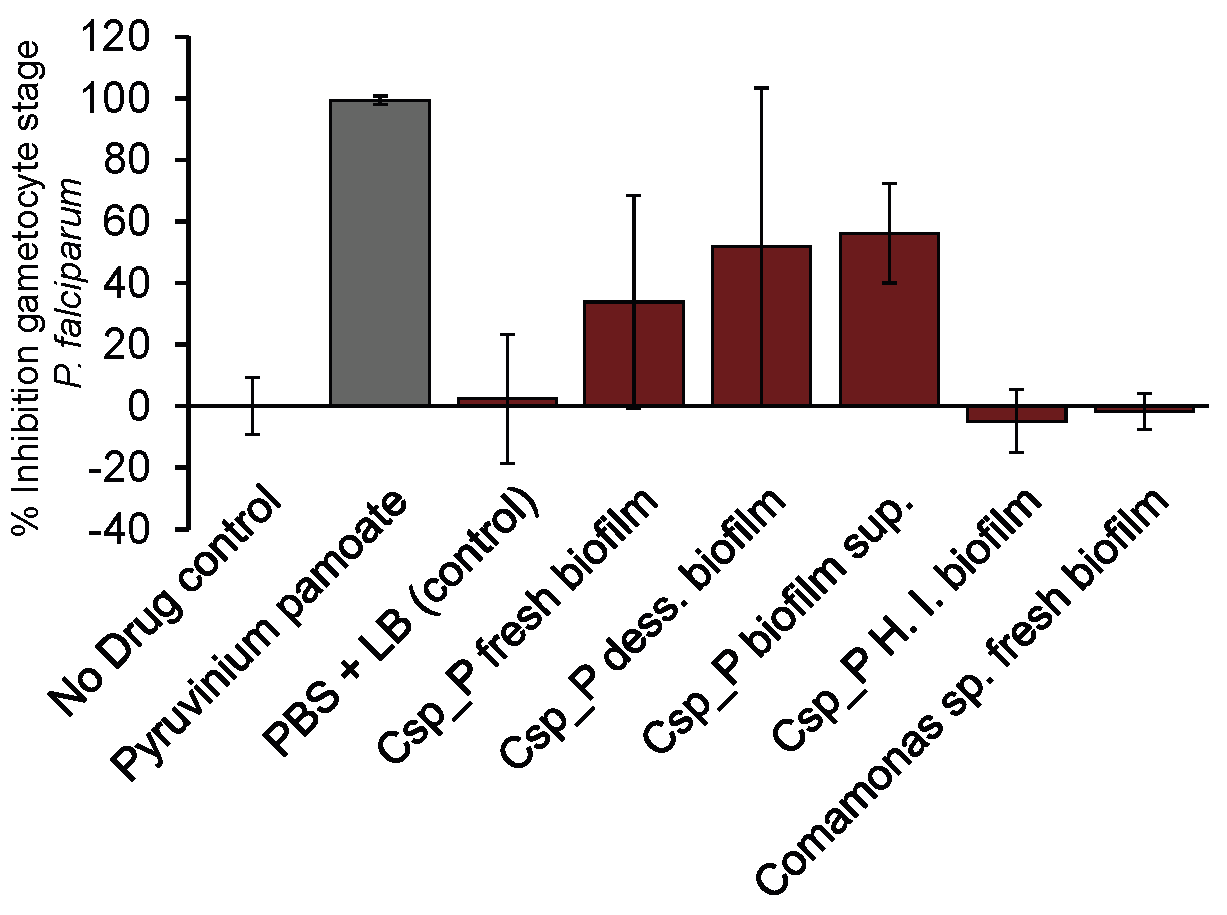

Supplement: Figure S2 — Effect of 36-h biofilm on gametocyte-stage P. falciparum . Csp_P cultures were filtered using a 0.2-µm filter and mixed with gametocyte-stage P. falciparum cultures. Erythrocytes were examined for gametocytes using Giemsa-stained blood films collected 3 days after Csp_P exposure. We determined gametocyte density per 1000 RBCs for each sample and performed a Tukey's test to determine whether each bacterial treatment significantly differed from the PBS+LB control. No treatments were significant, but biofilm 36-h supernatant trended toward significance (p = 0.06) (TIF) [file ppat.1004398.s003.tif]

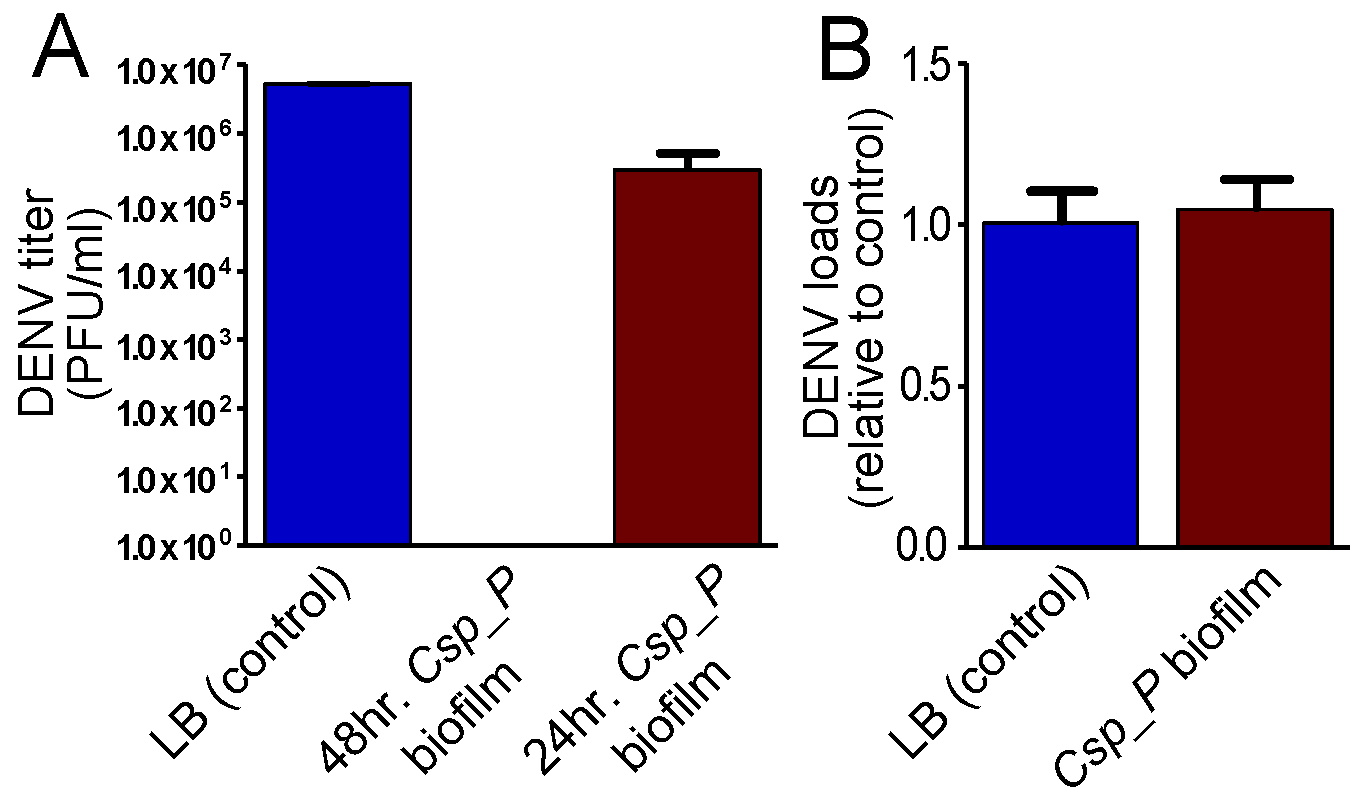

Supplement: Figure S3 — (A) Anti-dengue activity of fresh Csp_P biofilm is only weakly present after 24 h of growth at room temperature and becomes highly potent after 48 h of growth. Dengue virus was mixed 1∶1 with human blood and directly exposed to Csp_P biofilm grown for 24 or 48 h. Samples were incubated for 45 min and then collected, filtered, and used to infect C6/36 cells. (B) Dengue virus particles are not sequestered by Csp_P biofilm. We mixed dengue virus with Csp_P biofilm and incubated the mixture for 45 min. We then centrifuged samples and used qRT-PCR to quantify viral RNA in the supernatant of the experimental (biofilm+DENV) and control (LB+DENV) treatments. (TIF) [file ppat.1004398.s004.tif]

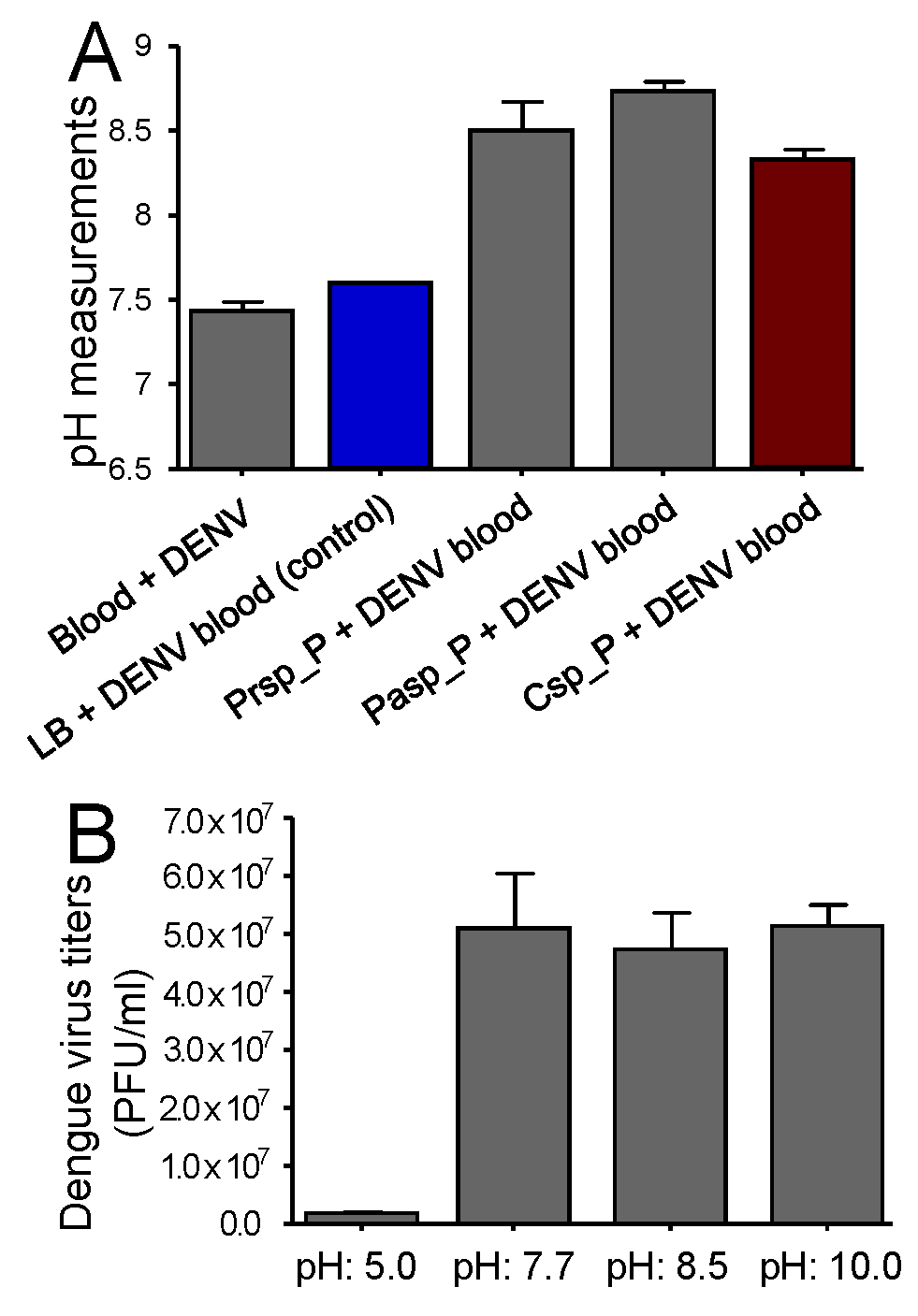

Supplement: Figure S4 — (A) Assessing changes in pH caused by Csp_P biofilm. We exposed dengue virus to Csp_P biofilm, incubated for 45 min, and measured the pH of the medium. (B) Assessing the effect of pH on dengue virus infectivity. We experimentally adjusted the pH of the MEM medium using NaOH and HCl to values of 5.0, 7.7, 8.5, and 10.0. We mixed the pH-adjusted media with dengue virus-laden human blood and incubated for 45 min., then collected and filtered the virus and used it to infect C6/36 cells. (TIF) [file ppat.1004398.s005.tif]

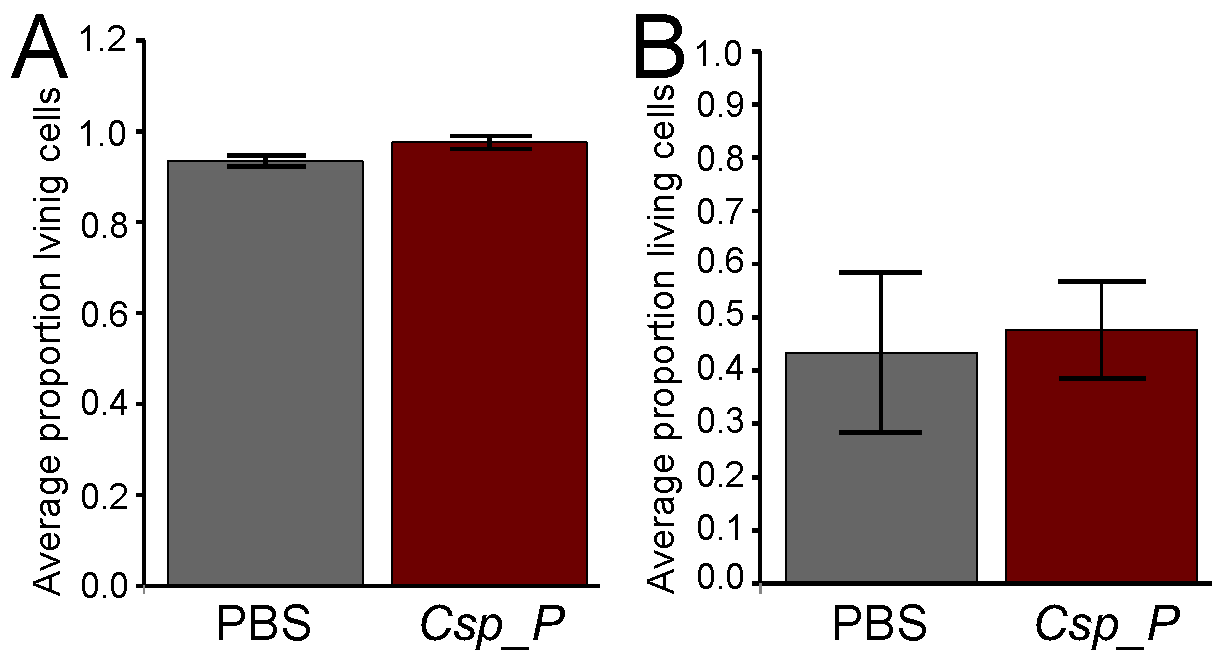

Supplement: Figure S5 — Crude biofilm extract does not have cytotoxic effects on insect or mammalian cells. We used trypan blue staining (0.4%, Invitrogen) to assay cell viability of BHK21-15 cells (A) and C6/36 cells (B) after a 45 min exposure to filtered Csp_P fresh biofilm. Difference in cell viability due to Csp_P exposure were non-significant for both cell lines (Mann Whitney Test). (TIF) [file ppat.1004398.s006.tif]

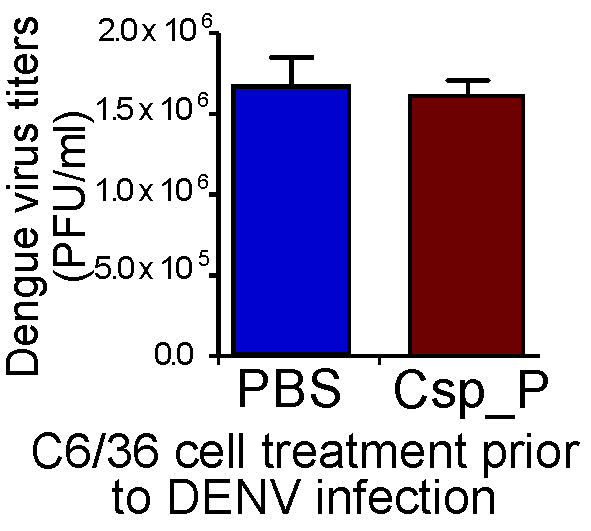

Supplement: Figure S6 — Exposure to Csp_P biofilm does not alter the insect cells' susceptibility to dengue virus. We filtered Csp_P biofilm using a 0.2-µm filter and exposed C6/36 cells (grown to 80% confluency) to the bacterial filtrate for 45 min. Csp_P biofilm filtrate was then washed from the cells using 1× PBS, and cells were infected with dengue virus. Cells were assessed for plaque formation at 6 days post-infection. (TIF) [file ppat.1004398.s007.tif]

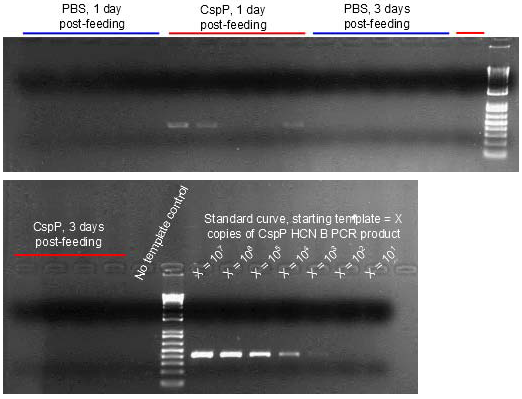

Supplement: Figure S7 — Representative gels from PCR diagnostic to assay presence of Csp_P in Aedes mosquito midguts. We used 10 ng of DNA from each Aedes female fed a sugar meal containing either PBS or Csp_P at a final concentration of 1010 CFU/ml. Using 10 ng of DNA from each sample as template, we performed a PCR using primers specific to the Csp_P hydrogen cyanide synthase B gene. (TIF) [file ppat.1004398.s008.tif]

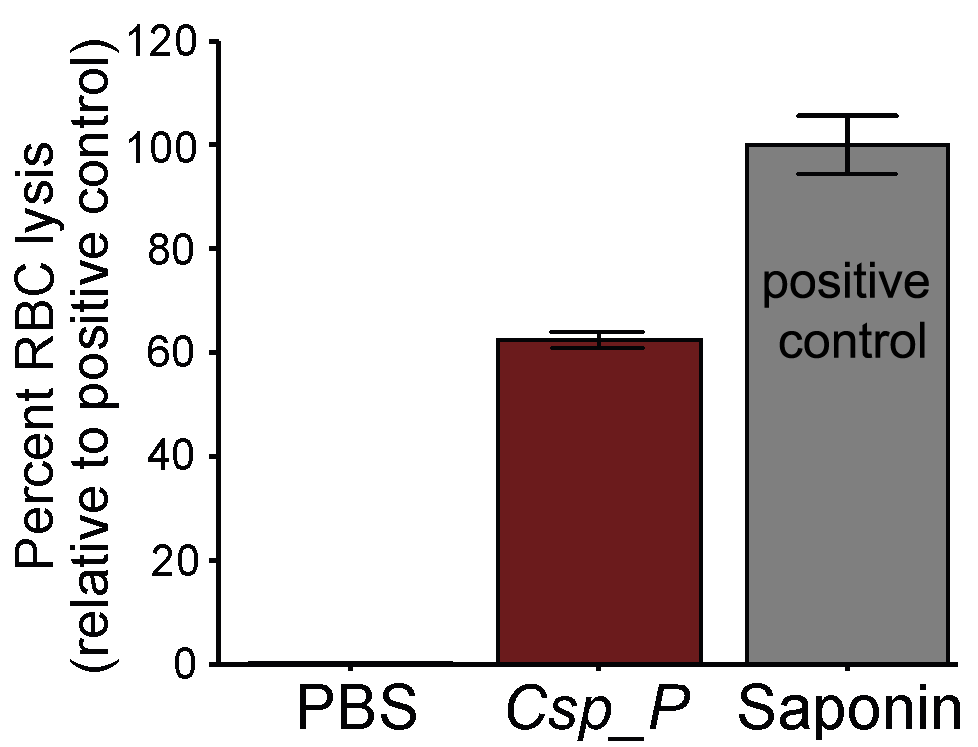

Supplement: Figure S8 — Csp_P biofilm is hemolytic when exposed to human red blood cells. We mixed filtered Csp_P fresh biofilm with human erythrocytes, incubated 24 h at 37°C and centrifuged at 2000 rpm for 5 min. We then removed the supernatant and assayed absorbance at 405 nm in an ELISA plate reader (HTS 7000 Perkin Elmer). 1× PBS was used as a negative control and saponin as a positive control. (TIFF) [file ppat.1004398.s009.tiff]
