# Supplementary material for: Chromobacterium Csp_P Reduces Malaria and Dengue Infection in Vector Mosquitoes and Has Entomopathogenic and In Vitro Anti-pathogen Activities
Source: PLoS Pathog. 2014 Oct 23;10(10):e1004398. doi: 10.1371/journal.ppat.1004398 (PMC4207801; doi:10.1371/journal.ppat.1004398)
Supplement: Table S1 — List of gene primers used in gene expression analyses of mosquito tissues post-bacterial challenge. (DOCX) [file ppat.1004398.s010.docx]

**Table S1 : List of gene primers used in gene expression analyses of mosquito tissues post-bacterial challenge.**

| **Species** | **Gene Name** | **Sequence** |
| --- | --- | --- |
|  |  |  |
| *Aedes aegypti* | Ribosomal S7 | Forward: 5'-GGGACAAATCGGCCAGGCTATC-3' |
|  |  | Reverse: 5'-TCGTGGACGCTTCTGCTTGTTG-3' |
|  |  |  |
|  | Defensin-C | Forward: 5'-TTGTTTGCTTCGTTGCTCTTT-3' |
|  |  | Reverse: 5'-ATCTCCTACACCGAACCCACT-3' |
|  |  |  |
|  | Cecropin-G | Forward: 5'-CCAAGCCTTGTGAACCAGTA-3' |
|  |  | Reverse: 5'-GGCCACCTGCTTCAGACT-3' |
|  |  |  |
|  | Cecropin-E | Forward: 5'-CGAAGCCGGTGGTCTGAAG-3' |
|  |  | Reverse: 5'-ACTACGGGAAGTGCTTTCTCA-3' |
|  |  |  |
|  | Lysozyme C | Forward: 5'-CCACGGCAACTGGATATGTCT-3' |
|  |  | Reverse: 5'-TCTGCGTCACCTTGGTGGTAT-3' |
|  |  |  |
|  |  |  |
| *Anopheles gambiae* | PGRP-LC | Forward: 5'-AGAATACCACACTAAGGCACAGT-3' |
|  |  | Reverse: 5'-AGACTTACGATCCTGGTAAATGT-3' |
|  |  |  |
|  | Cecropin 1 | Forward: 5'-CCAGAGACCAACCAACCACCAA-3' |
|  |  | Reverse: 5'-GCACTGCCAGCACGACAAAGA-3' |
|  |  |  |
|  | FBN9 | Forward: 5'-CCAAGATGTCGGGCAAGTAT-3' |
|  |  | Reverse: 5'-TTGTGGTACGTCAGCGAGTC-3' |
|  |  |  |
|  | TEP1 | Forward: 5'-ATGCTCTGCTGTCGTTTGTG-3' |
|  |  | Reverse: 5'-TTCGTGTCCTCCGGTATTTC-3' |
|  |  |  |
|  | LRRD7 | Forward: 5'-TCGGTGAGCAACAGTTTGA-3' |
|  |  | Reverse: 5'-CTTCATTCCCGCTAATGCT-3' |
|  |  |  |
|  | Defensin 1 | Forward: 5'-GCGGTTCCAAAGTTCCGACA-3' |
|  |  | Reverse: 5'-AGCGGGACACAAAATTGTTC-3' |
|  |  |  |
|  | Rel2 | Forward: 5'-CGGAGAAGTCGAAGAAAACG-3' |
|  |  | Reverse: 5'-CACAGGCACACCTGATTGAG-3' |
